# A Novel Magnetically Targeted Intramedullary (MagIC‐TI) Xenograft Model for Precise Leukemia Modeling and Drug Resistance Evaluation in the Bone Marrow Niche

**DOI:** 10.1155/jimr/3236026

**Published:** 2026-06-28

**Authors:** Qiusui Mai, Taosong Liu, Luxia Tang, Lihan Dai, Siyi Li, Jingyi Guo, Wen Xia, Lingxi Chen, Jiabao Wu, Jiawei Rong, Weishen Zhang, Jiaxing Zhang, Xiaojun Xu, Qianli Jiang

**Affiliations:** ^1^ Department of Blood Transfusion, The Seventh Affiliated Hospital, Sun Yat-sen University, Shenzhen, 518107, China, sysu.edu.cn; ^2^ First School of Clinical Medicine, Southern Medical University, Guangzhou, 510515, China, fimmu.com; ^3^ Southern Medical University, Guangzhou, 510515, China, fimmu.com; ^4^ Class of 2022, Eight-Year Clinical Medicine Program, Southern Medical University, Guangzhou, 510515, China, fimmu.com; ^5^ School of Stomatology, Southern Medical University, Guangzhou, 510515, China, fimmu.com; ^6^ Guangzhou Maishi Biotechnology Co., Ltd., Guangzhou, 510515, China; ^7^ Clinical Trial Center, Nanfang Hospital, Southern Medical University, Guangzhou, 510515, China, fimmu.com; ^8^ Department of Hematology, Nanfang Hospital, Southern Medical University, Guangzhou, 510515, China, fimmu.com

**Keywords:** acute myeloid leukemia (AML), bone marrow niche, drug resistance, minimal residues diseases (MRD), xenograft model

## Abstract

Acute myeloid leukemia (AML) remains a therapeutic challenge due to drug resistance and relapse, which are often driven by the protective bone marrow (BM) niche. Conventional xenograft models fail to adequately recapitulate this niche‐specific pathophysiology. To overcome this limitation, a novel magnetically targeted intramedullary (MagIC‐TI) xenograft model was developed. Magnetically labeled doxorubicin (DOX)‐resistant HL60 cells (Mag‐Re) were injected into the femurs of NSG (nonobese diabetic [NOD] Cg‐Prkdc^scid^IL2rg^tm1Wjl^/SzJ) mice using a patented microinjection syringe under localized magnetic guidance. With the MagIC‐TI model, rapid (day 1) and specific (100% by day 7) leukemic engraftment was achieved within the femoral BM, whereas intravenous (IV) injection led to delayed (mean 23.67 ± 10.26 days) and disseminated engraftment. Bioluminescence imaging, histopathological analysis, flow cytometry, and molecular assays confirmed that disease was localized in the MagIC‐TI model. In contrast, extramedullary infiltration, predominantly in the lungs, spleen, liver, and kidneys, was observed early in progression in the IV model. The MagIC‐TI model discriminated drug responses, showing effective tumor burden reduction with homoharringtonine (HHT) and unequivocal DOX resistance, a distinction that was obscured in heterogeneous IV models. Furthermore, employing a semisolid decalcification (SSD) system preserved green fluorescent protein (GFP) fluorescence, enabling high‐resolution visualization of engrafted cells within bone tissue. The MagIC‐TI model enables BM‐targeted, rapid, and efficient leukemic engraftment and allows discrimination of drug sensitivity and resistance. This model provides a robust and reproducible platform for modeling the leukemia BM niche and for preclinical evaluation of niche‐directed therapies.

## 1. Introduction

Acute myeloid leukemia (AML) remains a therapeutic challenge primarily due to drug resistance and relapse, which are driven by the protective bone marrow (BM) niche. Consequently, the 5‐year overall survival rate for adult patients is ~30% despite advances in chemotherapeutic and targeted agents [1–3]. Leukemia stem cells (LSCs) residing within the BM niche evade chemotherapy via direct interactions with stromal cells and soluble factor signaling, leading to minimal residual disease (MRD) persistence and subsequent relapse [4–6]. However, conventional preclinical models fail to adequately recapitulate this niche‐dependent pathophysiology, limiting progress in developing effective therapies.

Conventional intravenous (IV) xenograft models, the current standard for AML research, exhibit critical limitations: delayed and heterogeneous engraftment, with leukemic cells often disseminating to extramedullary sites (e.g., lungs and spleen) before colonizing the BM [7, 8]. As a result, these models fail to recapitulate the BM niche’s protective effects, leading to inaccurate assessments of drug efficacy and resistance mechanisms [9–12]. The absence of a physiologically relevant model that recapitulates niche‐mediated resistance has hindered the translation of preclinical findings into clinical practice.

Pioneering work by our research group has established a strong foundation for targeted cell delivery approaches. Mai et al. [13] demonstrated that magnetic targeting could significantly enhance the retention of BM cells within the femoral niche while accelerating hematopoietic reconstitution. In a more direct precursor to the current study, Zhang et al. [14] developed a magnetic micro‐living‐motor (MLM) system that achieved a targeted colonization efficiency of 82.76% for tumor cells in the femoral marrow, outperforming conventional delivery methods. Building on this system, the present study utilized the MLM platform to investigate niche‐mediated drug resistance and leukemia progression.

Leveraging these advances, a novel magnetically targeted intramedullary (MagIC‐TI) xenograft model was developed to enhance the homing and retention of doxorubicin (DOX)‐resistant AML cells within the BM niche. This model promotes efficient intramedullary engraftment and recapitulates the scenario of MRD persistence in protective BM microenvironments.

To enable comprehensive longitudinal monitoring, bioluminescence imaging, histopathological analysis, flow cytometry, and molecular detection of WT1 expression were integrated. Key technical components included magnetic targeting and intra‐BM injection via a patented microinjection syringe (Patent Number CN201620090904.2) and the semisolid decalcification (SSD) system. A critical technical barrier to studying BM‐resident leukemia is visualizing cells within the hard, opaque bone. To overcome this, a proprietary SSD system was employed that preserves green fluorescent protein (GFP) fluorescence in situ after decalcification and sectioning, enabling high‐resolution visualization of donor cells within the bone matrix [15–17].

The present study aimed to (1) establish a MagIC‐TI model that recapitulates niche‐dependent AML pathophysiology; (2) compare its performance with that of conventional IV injection with respect to engraftment efficiency, disease progression, and drug response; and (3) validate its utility for evaluating drug efficacy and resistance. By overcoming the spatial and temporal constraints of existing models, the MagIC‐TI system offers a robust platform for studying niche‐mediated resistance mechanisms and accelerating the development of novel anti‐leukemic strategies targeting the BM niche.

This work represents an advance in preclinical AML modeling, offering a more physiologically relevant tool to bridge the gap between in vitro discovery and clinical translation.

## 2. Materials and Methods

### 2.1. Generation and Characterization of Magnetically Labeled HL60‐ADR‐GL Cells

DOX‐resistant HL60 cells (HL60‐ADR) (Laboratory of Hematology, Nanfang Hospital, Southern Medical University) were stably transduced with lentiviral vectors encoding enhanced GFP (eGFP) and luciferase, then sorted via fluorescence‐activated cell sorting (FACS, BD FACSAria III, BD Biosciences, San Jose, CA, USA) to establish the HL60‐ADR‐GL cell line. Gene transduction efficiency (Figure S1) and DOX sensitivity (Figure S2) of HL60‐ADR‐GL were validated prior to experiments.

**Figure 1:**
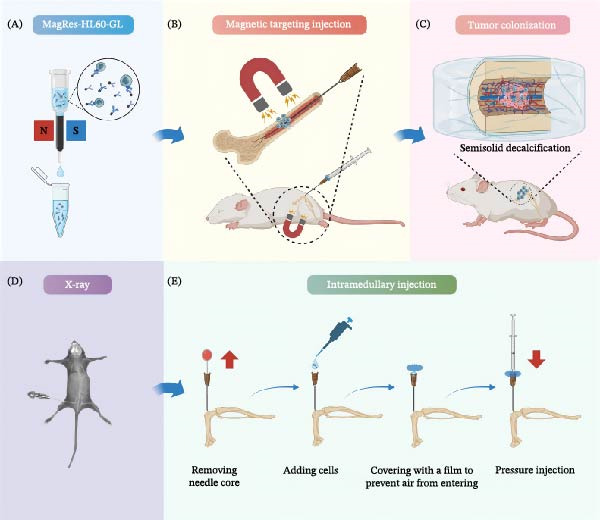
Schematic diagram of the MagIC‐TI system to construct a precision engraftment murine model. (A) Schematic diagram of CD33‐conjugated magnetic microbeads labeled HL60 cells. (B) Magnetic targeted injection into femur under the action of an applied magnetic field. (C) Precision engraftment and colonization in femur which can be observed under confocal with the use of the semisolid decalcification system. (D) X‐ray of successful intramedullary injection. (E) The method of intramedullary injection using a bone marrow microinjector containing a syringe and a needle core invented by our team (Patent Number 201620090904.2).

For magnetic labeling, HL60‐ADR‐GL cells (1 × 10^7^ cells/80 μL PBS, 20 μL microbeads) were incubated with CD33 MicroBeads (130‐045‐501; Miltenyi Biotec, Bergisch Gladbach, Germany) at 2–8°C for 20 min according to the manufacturer’s protocol. After incubation, cells were washed with PBS and subjected to magnetic separation using an MS MACS column (130‐042‐201) on a MiniMACS Separator (130‐042‐102; Miltenyi Biotec) to yield magnetically labeled HL60‐ADR‐GL cells (MagRe) (Figure 1A). The magnetic selection rate was calculated as (Cell count after selection/total cell count before selection) × 100%.

To confirm the phenotypic stability of magnetized HL60‐ADR‐GL cells, surface expression of CD45 (anti‐human CD45‐APC, clone HI30, BioLegend, San Diego, CA, USA) and CD33 (anti‐human CD33‐PerCP‐Cy5.5, clone WM53, eBioscience, San Diego, CA, USA) was assessed by flow cytometry. The morphology, immunophenotype, proliferation, apoptosis, migration, aggregation, and gene expression of magnetically labeled (MagRe) and unlabeled (Non‐Mag) HL60‐ADR‐GL cells were subsequently characterized.

The cellular ultrastructure was examined by transmission electron microscopy (TEM). For scanning electron microscopy (SEM), 1 × 10^9^ cells per group were fixed in 2.5% glutaraldehyde, mounted onto aluminum stubs, and examined with an S‐3000N microscope (Hitachi, Tokyo, Japan) at the Electron Microscopy Core Facility of Southern Medical University (*n* = 5). For quantitative assessment, 100 randomly selected cells per group were evaluated. Cells were scored as “intact” if they exhibited a continuous plasma membrane, a normal nuclear morphology, and the absence of cytoplasmic vacuolation or organelle swelling.

To evaluate the proliferative capacity of magnetically labeled cells, MagRe and Non‐Mag cells were seeded in 96‐well plates at a density of 0.5 × 10^5^ cells/well in 100 μL of complete RPMI‐1640 medium containing 10% FBS (*n* = 5 wells per group per time point). The cells were cultured for up to 7 days. To avoid nutrient depletion and pH changes, the medium was half‐replaced with a fresh complete medium every 48 h. On days 1, 2, 3, 4, 5, 6, and 7, 10 μL of CCK‐8 solution (CK04‐11, Dojindo Laboratories) was added to each well, and the plates were incubated for an additional 3 h at 37°C. The absorbance at 450 nm (OD_450_) was measured using a microplate reader (SpectraMax iD3, Molecular Devices).

For apoptosis analysis, cells from the MagRe and Non‐Mag groups were cultured under identical conditions for up to 28 days (*n* = 5 replicates per group per time point). At days 0, 7, 14, 21, and 28, cells were harvested and stained with an annexin V‐APC/PI Apoptosis Kit (E‐CK‐A217, Elabscience). The percentage of apoptotic cells (annexin V+) was determined using a BD FACSCanto II flow cytometer (BD Biosciences) and analyzed with FlowJo software (v10.10).

### 2.2. Quantitative Real‐Time PCR for Apoptosis‐, Ferroptosis‐, and Homing‐Related Genes

To preliminarily assess whether CD33‐mediated magnetic labeling affects the expression of genes involved in apoptosis (BAX and CASP3), ferroptosis (GPX4 and ACSL4), and homing (CXCR4 and MAPK1), MagRe and Non‐Mag cells were cultured in parallel for 48 h after magnetic selection. Total RNA was extracted from 1 × 10^6^ cells per group (*n* = 5 replicates/group) using the TRIzol reagent (Invitrogen, Carlsbad, CA, USA), and 1 μg of RNA was reverse‐transcribed using PrimeScript RT Master Mix (Takara Bio, Kusatsu, Japan). Quantitative PCR was performed using TB Green Premix Ex Taq II (Takara Bio) on a CFX96 Real‐Time PCR Detection System (Bio‐Rad, Hercules, CA, USA). Primer sequences are provided in Table S1.

### 2.3. Magnetic Field‐Directed Migration and Aggregation Assays

To evaluate the magnetism‐induced targeted capability of MagRe cells, migration and aggregation assays were performed under a magnetic field.

For the antigravity migration assay, cells (1 × 10^5^/well) were seeded into 35‐mm Petri dishes filled with a complete medium and allowed to contact the lid. After 24 h, adherent cells on the lid were observed under a fluorescence microscope.

For the Transwell migration assay, cells (1 × 10^5^/well) were seeded onto Matrigel‐precoated Transwell inserts (8‐μm pores) placed in 24‐well plates containing a complete medium in the lower chamber. A magnetic field was applied beneath the wells for 24 h. Nonmigrated cells on the upper membrane surface were removed, and migrated cells on the lower surface were photographed and counted.

For the aggregation assay, MagRe and Non‐Mag were resuspended at 1 × 10^6^ cells/mL, and 1 mL was placed into each well of a 12‐well plate (*n* = 5). A neodymium magnet (260 mT) was attached to the bottom center of each well. After 24 h of incubation, the magnet was removed, and cell aggregation was observed under an inverted fluorescence microscope.

### 2.4. Mice

Male nonobese diabetic (NOD) Cg‐Prkdc^scid^IL2rg^tm1Wjl^/SzJ (NSG) mice (6–8 weeks old) were purchased from Jiangsu Jicui Yaokang Biotechnology Co., Ltd. and maintained in a specific pathogen‐free (SPF) barrier system at the Experimental Animal Center of Nanfang Hospital, Southern Medical University. Male mice were used in this initial study to avoid potential confounding effects of the estrous cycle on leukemia engraftment and progression, as previously reported [18, 19]. All animal procedures were approved by the Experimental Animal Center of Southern Medical University (Approval Number IACUC‐LAC‐20241010‐007).

### 2.5. Construction of Magnetically Targeted Leukemia Cell Model

For comparison of the magnetically targeted and conventional IV models, 30 mice were randomly assigned to three groups (*n* = 10/group): the MagIC‐TI group, the IV group, and wild‐type (WT). Following anesthesia with pentobarbital sodium (0.2–0.3 mL of 0.3% w/v PBS, intraperitoneal), the MagIC‐TI group received an intramedullary injection of 5 × 10^6^ MagRe cells in 30 μL PBS using a custom BM aspiration needle (Patent Number CN201620090904.2) under a precision magnetic field, which was maintained for 2 h postinjection. The IV group was administered 5 × 10^6^ Non‐Mag cells in 100 μL PBS via the tail vein, and the WT group received an equal volume of PBS through intramedullary injection. At prespecified time points, hematological parameters, flow cytometry, histopathological examination, BM morphology analysis, and WT1 gene load were performed. Experiments were replicated under identical conditions to monitor survival, body weight changes, general status, and bioluminescence imaging.

### 2.6. Drug Resistance Evaluation

MagIC‐TI and IV leukemia models were established, as described above. Three days after confirming successful engraftment via bioluminescence imaging, mice from each model were randomly allocated into three treatment groups (*n* = 10/group): DOX (2 mg/kg), HHT (homoharringtonine, 1 mg/kg), and Ctrl (vehicle control). Treatment was initiated when the average bioluminescence signal intensity reached >1 × 10^6^ photons/s, which corresponded to day 7 for the MagIC‐TI model and day 21 for the IV model. Drugs or PBS (200 μL) were administered intraperitoneally once daily for three consecutive days. Treatment response was evaluated by serial bioluminescence imaging, flow cytometry, histopathological examination of BM, and MRD analysis via quantitative PCR for WT1 expression 7 days after treatment.

### 2.7. Bioluminescence Imaging

Bioluminescence imaging was performed to monitor the tumor burden in vivo using an IVIS imaging system (Ami HTX, Spectral Instruments Imaging, USA). Mice were injected intraperitoneally with 100 µL of D‐luciferin potassium salt (10 mg/mL, PerkinElmer, USA) and imaged after 5–10 min under standardized parameters (exposure time = 60 s, f/stop = 1.2, and field of view = 25 × 17 cm). The bioluminescence intensity within the region of interest (ROI) was quantified using Aura software (Spectral Instruments Imaging). For ex vivo imaging, mice received an identical injection of D‐luciferin, after which hind limbs and selected organs were rapidly dissected within 5–10 min and placed in the imaging chamber for signal acquisition with the same system and software.

### 2.8. Histopathological Analysis

Lung, spleen, liver, and kidney tissues were fixed in 4% paraformaldehyde and processed for cryosectioning at 20 μm. The injected femur was fixed in 4% paraformaldehyde for 24 h and then bisected longitudinally at the midpoint to expose the marrow cavity. Bone segments were embedded in a SSD system containing 0.1 g/mL EDTA, 0.004 g/mL agarose, and 5% (v/v) formic acid in phosphate‐buffered saline (PBS, pH ≥ 4), ensuring full infiltration of the medullary cavity. The SSD system was maintained at room temperature in the dark for 4 weeks, after which 20‐μm cryosections were prepared.

### 2.9. Flow Cytometry Analysis of Tumor Burden

Peripheral blood and BM samples were filtered through a 70 μm cell strainer (352350, Falcon) to obtain single‐cell suspension, followed by red blood cell lysis (00‐4300‐54, eBioscience) at 4°C. Cell suspension was washed, and tumor burden (percentage of GFP^+^) was evaluated by flow cytometry (BD FACSCanto II).

### 2.10. Quantitative Real Time PCR (RT‐qPCR)

In the leukemia cell model, WT1 expression was measured as a molecular marker. At prespecified time points (days 7 and 10), 100 μL of peripheral blood or tissue was collected. Total RNA was extracted, and RT‐qPCR was performed for detection. β‐Actin served as the reference gene, and the relative mRNA expression levels were compared using the 2^−∆∆Ct^ method. Primer sequences for signature genes were as followed: WT1‐F: AGCGACGAGAAGAGCTACAAG; WT1‐R: GGGTTTGCATGGTTTCCAG; β‐actin‐F: CACGAAACTACCTTCAACTCCATC; β‐actin‐R: AGCACTGTGTTGGCGTACAG.

### 2.11. Statistical Analysis

Statistical analyses were performed using GraphPad Prism 9.0 (GraphPad Software, San Diego, CA, USA) and IBM SPSS Statistics 21 (IBM Corp., Armonk, NY, USA). Quantitative data are presented as mean ± standard deviation (SD), as specified in the figure legends. Comparisons between two groups were analyzed using a two‐sided unpaired Student’s *t*‐test. For comparisons involving more than two groups, one‐way analysis of variance (ANOVA) followed by Tukey’s post hoc test was applied. For longitudinal data with repeated measurements over time, two‐way repeated‐measures ANOVA was used to assess the effects of time, group, and their interaction. Tumor incidence rates were compared using Pearson’s chi‐square test, and survival curves were analyzed by the Kaplan–Meier method with the log‐rank test. All experiments included at least three independent biological replicates or the number of animals indicated in the figure legends. Statistical significance was defined as follows: ns, not significant (*p* > 0.05);  ^∗^ *p*  < 0.05;  ^∗∗^ *p*  < 0.01;  ^∗∗∗^ *p*  < 0.001;  ^∗∗∗∗^ *p*  < 0.0001.

## 3. Results

### 3.1. Characterization of Magnetically Labeled HL60 Cells (MagRe‐HL60‐GL)

To ensure that the magnetic labeling process did not compromise the fundamental biological properties of the leukemia cells, thereby ensuring the reliability of the subsequent animal model, a systematic characterization of MagRe‐HL60‐GL cells was first performed.

**Figure 2:**
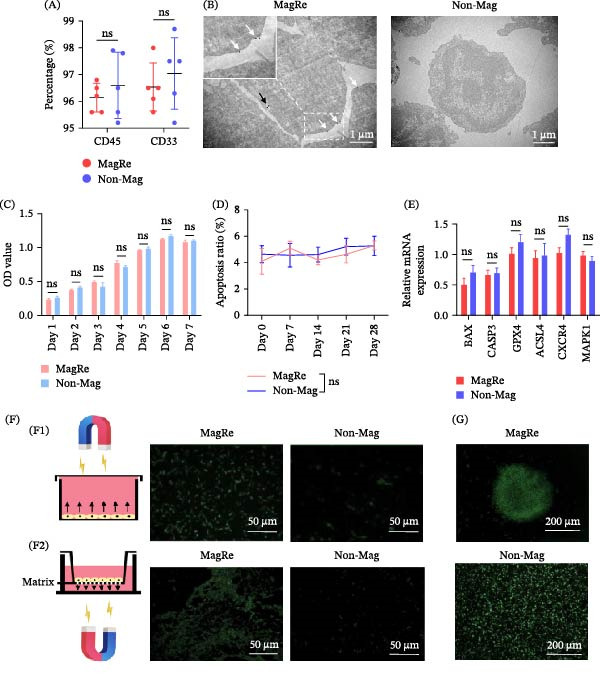
Characterization of magnetically labeled HL60‐ADR‐GL cells (MagRe) in vitro. (A) Flow cytometric analysis of CD45 and CD33 surface expression in MagRe and unlabeled HL60‐ADR‐GL (Non‐Mag) cells. (B) Representative TEM images showing CD33‐conjugated magnetic microbeads as electron‐dense nanoparticles attached to the cell membrane surface (white arrows) or internalized within phagocytic vesicles (black arrows). Scale bar: 1 μm. (C) Proliferation kinetics assessed by CCK‐8 assay over 7 days. (D) Apoptosis assessment by annexin V‐APC/PI staining over 28 days. (E) Relative mRNA expression of apoptosis‐related (BAX, CASP3), ferroptosis‐related (GPX4, ACSL4), and homing‐related (CXCR4, MAPK1) genes after 48 h of culture without magnetic field. Data are normalized to β‐actin. (F) Magnetic field‐directed migration assays. (F1) Antigravity migration assay showing semiadherent MagRe cells on the lid compared to Non‐Mag controls. (F2) Transwell migration assay through Matrigel‐coated membranes. Scale bars: 50 μm. (G) Aggregation behavior under a localized magnetic field for 24 h. Representative inverted fluorescence microscopy images show multicellular cluster formation in MagRe cells, whereas Non‐Mag cells remained dispersed. Scale bar: 200 μm. Data information: all quantitative data are presented as mean ± SD with *n* = 5 replicates/group. Statistical analysis was performed using two‐sided Student’s *t*‐test for (A) and (E), and two‐way repeated‐measures ANOVA for (C) and (D).  ^∗^ *p* < 0.05,  ^∗∗^ *p* < 0.01,  ^∗∗∗^ *p* < 0.001,  ^∗∗∗∗^ *p* < 0.0001; ns, not significant.

The magnetic selection using CD33 MicroBeads under optimized protocols yielded a selection rate of 85.78% ± 9.11% (*n* = 5 replicates). Phenotypic characterization confirmed stable, high surface expression of both CD45 (MagRe vs. Non‐Mag: 96.14% ± 0.48% vs. 96.60% ± 1.10%, *p*  > 0.05) and CD33 (MagRe vs. Non‐Mag: 96.54% ± 0.80% vs. 97.04% ± 1.19%, *p*  > 0.05) (Figure 2A and Figure S3).

TEM was employed to visualize the intracellular distribution of the magnetic nanoparticles. As shown in Figure 2B, CD33‐conjugated microbeads were observed as electron‐dense nanoparticles attached to the cell membrane surface or internalized within phagocytic vesicles. To quantitatively assess the impact on the cellular ultrastructure, TEM images were analyzed from 100 randomly selected cells per group (*n* = 5 replicates/group). The percentage of cells displaying an intact plasma membrane, normal nuclear morphology, and absence of cytoplasmic vacuolation was calculated. MagRe cells showed a high proportion of intact morphology (94.30% ± 2.10%), comparable to unlabeled HL60‐ADR‐GL cells (95.70% ± 1.80%) (MagRe vs. Non‐Mag: *p* > 0.05).

To determine whether magnetic labeling affected the proliferative capacity, CCK‐8 assays were performed over 7 days. Both MagRe and Non‐Mag cells exhibited similar growth kinetics, with OD_450_ values increasing progressively from day 1 to day 6 and plateauing on day 7. No significant difference was observed between the two groups at any time point (*p* > 0.05, *n* = 5 replicates per group per time point) (Figure 2C).

To evaluate potential delayed pro‐apoptotic effects, MagRe and Non‐Mag cells were cultured for 28 days, and apoptosis was assessed at the indicated time points by annexin V‐APC/PI staining. The percentage of apoptotic cells remained low and comparable between groups throughout the culture period (MagRe: 4.10% to 5.30%; Non‐Mag: 4.56% to 5.27%, *p* > 0.05, *n* = 5 replicates per group per time point) (Figure 2D, with gating strategy shown in Figure S4).

### 3.2. Expression of Selected Apoptosis‐, Ferroptosis‐, and Homing‐Related Genes In Vitro

A preliminary comparison of mRNA levels of apoptosis‐related genes (BAX, CASP3), ferroptosis‐related genes (GPX4, ACSL4), and homing‐related genes (CXCR4, MAPK1) between MagRe‐HL60‐GL and unlabeled HL60‐ADR‐GL cells after 48 h of culture without a magnetic field revealed no significant differences for any of the six genes examined (all *p*  > 0.05, *n* = 5 replicates/group) (Figure 2E).

### 3.3. In Vitro Evaluation of Magnetic Field‐Directed Migration and Aggregation of Labeled HL60 Cells

The MagIC technology enabled targeted migration of MagRe cells. In the antigravity migration assay (Figure 2F1), numerous semiadherent, spindle‐shaped MagRe cells grew on the lid, whereas few Non‐Mag cells adhered (326 ± 24 vs. 3 ± 2 per field, *n* = 5 replicates/group, *p*  < 0.001). In the Transwell migration assay (Figure 2F2), significantly more MagRe cells migrated through the Matrigel‐coated membrane compared to Non‐mag group (174 ± 22 vs. 6 ± 1 per field, *n* = 5 replicates/group, *p* < 0.001). Moreover, when exposed to a localized magnetic field for 24 h, MagRe cells rapidly assembled into multicellular clusters around the magnetic source, whereas nonmagnetized HL60‐ADR‐GL cells remained evenly dispersed and showed no obvious aggregation (Figure 2G).

### 3.4. Bioluminescence Imaging of Tumor Engraftment and Incidence

**Figure 3:**
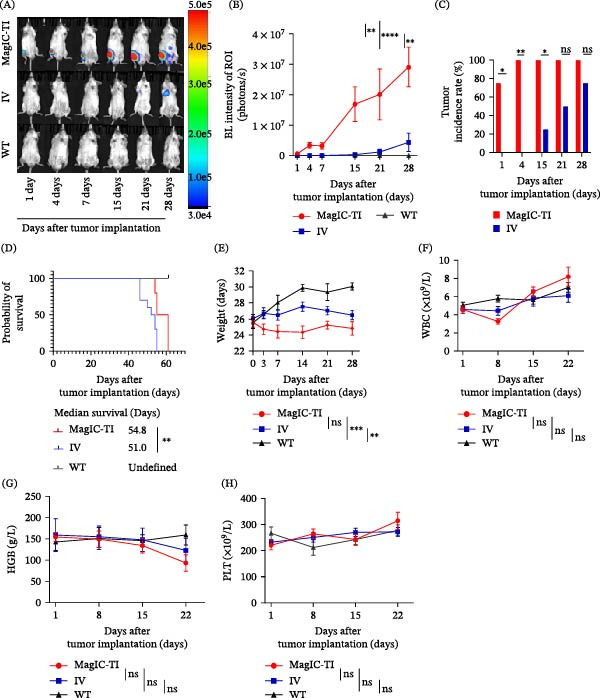
Tumor engraftment, systemic progression, and general condition of leukemia‐bearing mice. (A, B) Representative bioluminescence images (A) and quantification of bioluminescence intensity in the region of interest (B) for MagIC‐TI, IV, and WT groups at indicated days after transplantation. (C) Tumor incidence rates from day 1 to day 28 postimplantation. (D) Survival curves of the three groups. (E) Body weight changes from day 0 to day 28 post transplantation. (F–H) White blood cell (WBC) counts (F), hemoglobin (HGB) levels (G), and platelet (PLT) counts (H) from day 1 to day 22 post transplantation. Data information: quantitative data are presented as mean ± SD with *n* = 10 mice per group. For (B) and (E–H), two‐way repeated‐measures ANOVA followed by Bonferroni post hoc test was used for multiple between‐group comparisons at each time point. For (C), Pearson’s chi‐square test was applied for comparisons of tumor incidence between groups. Survival analysis (D) was performed using the Kaplan–Meier method with log‐rank test.  ^∗^ *p* < 0.05,  ^∗∗^ *p* < 0.01,  ^∗∗∗^ *p* < 0.001,  ^∗∗∗∗^ *p* < 0.0001; ns, not significant.

BLI revealed distinct tumor signal patterns between the two groups (Figure 3A). In the MagIC‐TI group, tumor signals localized specifically to the injected femur as early as day 1 postimplantation. In contrast, the IV group exhibited weak and unstable fluorescence signals in the lungs, with no consistent or stable localization until day 21 postinjection, with an average tumor formation time of 23.67 ± 10.26 days. (Figure 3A). Quantitative ROI analysis revealed a significantly higher overall signal intensity curve in the MagIC‐TI group than in the IV group (*n* = 10 mice/group, *p*  < 0.01) (Figure 3B). Tumor incidence rates also differed significantly between the two groups (Figure 3C), with the MagIC‐TI group achieving 100% femoral engraftment by day 7, whereas the IV group showed significantly lower and delayed engraftment (Figure 3C).

### 3.5. General Condition and Hematological Characteristics

Survival analysis demonstrated significantly prolonged survival in the MagIC‐TI model compared with the IV model (*n* = 10 mice/group, *p* = 0.0024) (Figure 3D). In the MagIC‐TI group, the first death occurred on day 54, and the last mouse was euthanized on day 61 due to >20% body weight loss. The IV group exhibited earlier mortality, with the first death on day 46 and all animals succumbing by day 55.

The MagIC‐TI group maintained a higher body weight than the IV group throughout the observation period, although the difference was not significant (*n* = 10 mice/group, *p*  > 0.05) (Figure 3E). White blood cell (WBC) counts increased in both MagIC‐TI and IV groups at day 8 compared with the WT group, but the differences did not reach statistical significance (*n* = 10/group, *p*  > 0.05) (Figure 3F). As the disease progressed, both tumor‐bearing groups showed a similar downward trend in hemoglobin (HGB) levels (Figure 3G). No significant differences in HGB or platelet (PLT) counts (Figure 3H) were observed among the three groups at any time point (*n* = 10 mice/group, *p*  > 0.05).

### 3.6. Integrated BLI, Histopathology, Flow Cytometric, and Molecular Profiling of Leukemic Engraftment

Bioluminescence imaging, histopathology, flow cytometry, and molecular assays were combined to quantitatively assess the tumor burden and distribution in tissues and organs on day 21 posttransplantation.

**Figure 4:**
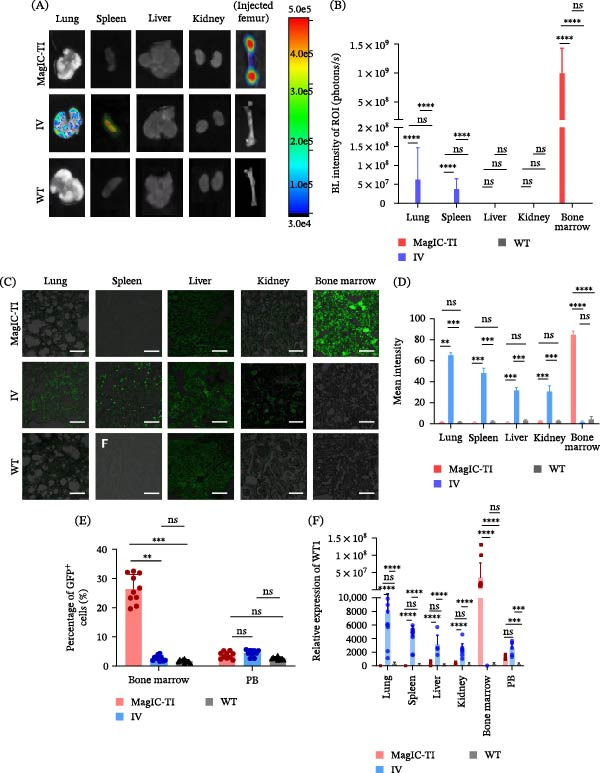
Integrated ex vivo bioluminescence, histopathological, flow cytometric, and molecular profiling of leukemic engraftment in multiple organs and tissues at day 21 posttransplantation. (A, B) Representative ex vivo bioluminescence images (A) and quantification (B) of bioluminescence signals in the lung, spleen, liver, kidney, and injected femur from MagIC‐TI, IV, and WT groups. (C, D) Representative confocal fluorescence microscopy images (C) and quantification (D) of tissue sections from the indicated organs and injected femur in the three groups. (E) Flow cytometric analysis of GFP^+^ tumor cells in the injected femoral bone marrow and peripheral blood. (F) WT1 mRNA expression levels in various tissues measured by RT‐qPCR. Data information: all quantitative data are presented as mean ± SD with *n* = 10 mice per group. For (B), (D), (E), and (F), one‐way ANOVA followed by Tukey’s post hoc test was used for multiple comparisons among the MagIC‐TI, IV, and WT groups.  ^∗^ *p* < 0.05,  ^∗∗^ *p* < 0.01,  ^∗∗∗^ *p* < 0.001,  ^∗∗∗∗^ *p* < 0.0001; ns, not significant.

Ex vivo imaging of isolated bones and organs revealed that in the MagIC‐TI group, tumor signals were specifically localized to the injected femur, with minimal signals in peripheral organs. In the IV group, signals were primarily detected in the lungs and spleen, with no femoral signal. No BLI signal was detected in the liver or kidney in either group (*n* = 10 mice/group) (Figure 4A,B).

Confocal fluorescence microscopy further resolved the spatial distribution. Consistent with BLI results, GFP^+^ cells were exclusively observed in the injected femur of MagIC‐TI mice. In the IV group, abundant GFP^+^ cells were present in the lungs and spleen, and sparse GFP^+^ cells were also detected in the liver and kidney; no GFP fluorescence was observed in the femoral BM (*n* = 10 mice/group) (Figure 4C,D). Histopathological examination by hematoxylin and eosin staining of tissue sections and Wright–Giemsa staining of BM smears confirmed these distinct engraftment patterns, with minimal extramedullary infiltration and hypercellular, blast‐dominated marrow in the MagIC‐TI group, in contrast to extensive leukemic infiltration in the lung, spleen, and liver and relatively normal marrow morphology in the IV group (Figure S5).

On day 21, flow cytometry of the injected femoral BM in the MagIC‐TI group identified a distinct population of GFP^+^ tumor cells, confirming successful local engraftment. Few GFP^+^ cells were detected in the peripheral blood of either group at this stage (*n* = 10 mice/group, Figure 4E) (Gating strategy shown in Figure S6).

Corroborating these findings, qPCR for the WT1 expression showed high levels specifically in the femoral BM of the MagIC‐TI group. In the IV group, WT1 expression was significantly higher in the lung, spleen, liver, and kidney compared with the MagIC‐TI group, mirroring the histopathological evidence of extensive extramedullary involvement. Minimal WT1 expression was observed in peripheral blood (*n* = 10/group) (Figure 4F).

Collectively, these data demonstrate that the MagIC‐TI model achieves robust, localized BM engraftment, whereas the IV model results in early, disseminated extramedullary disease.

### 3.7. The MagIC‐TI Model Enables Precise Assessment of Drug Response and Resistance

The translational utility of the MagIC‐TI platform for evaluating drug efficacy and detecting resistance was subsequently assessed. To standardize treatment initiation, the therapeutic window was defined as the interval from the time when the bioluminescence signal intensity first exceeded 1 × 10^6^ photons/s (window onset) to the time of death (mean survival). The MagIC‐TI model provided a substantially wider therapeutic window (day 7 to day 54 posttransplantation) compared with the IV model (day 21 to day 51 posttransplantation). In both models, the tumor burden was evaluated 1 week after treatment initiation using BLI, flow cytometry, histopathological examination of BM, and qPCR for WT1 expression.

**Figure 5:**
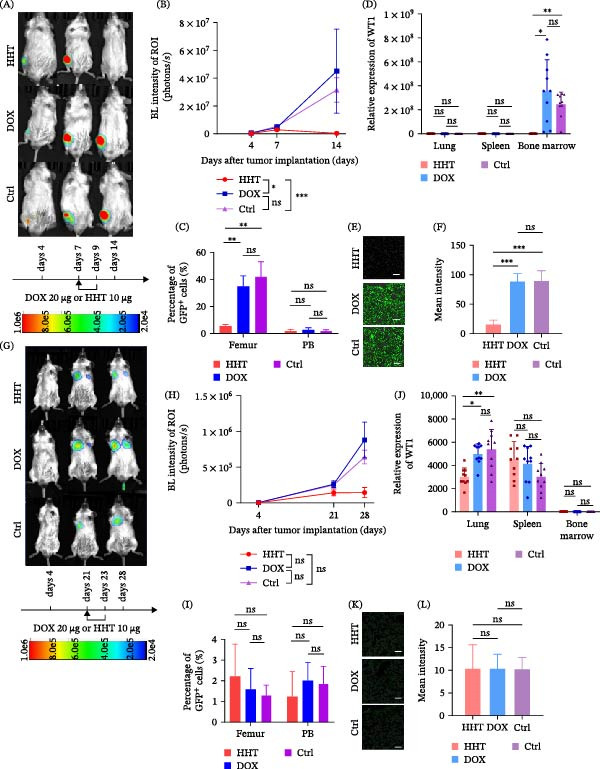
The MagIC‐TI model enables precise assessment of drug response and resistance, whereas the IV model yields inconclusive results. Mice bearing established leukemia (BLI signal >1 × 10^6^ photons/s) were treated with doxorubicin (DOX, 2 mg/kg), homoharringtonine (HHT, 1 mg/kg), or vehicle control (Ctrl) for three consecutive days. Tumor burden was evaluated 7 days after treatment. (A–F) MagIC‐TI model. (A) Representative bioluminescence images showing signals strictly localized to the injected femur. (B) Quantitative ROI analysis of BLI signals. (C) Flow cytometric quantification of GFP^+^ cells in bone marrow. (D) WT1 mRNA expression in bone marrow by qPCR. (E, F) Representative GFP fluorescence images of bone marrow tissue sections (E) and corresponding quantification (F). Scale bars: 50 µm. (G–L) IV model. (G) Representative BLI images showing disseminated signals. (H) ROI quantification of BLI signals. (I) Flow cytometry of GFP^+^ cells in bone marrow. (J) WT1 expression in lung, spleen, and bone marrow. (K,L) Representative GFP expression images in bone marrow sections (K) and corresponding quantification (L). Scale bars: 50 µm. Data information: all quantitative data are presented as mean ± SD with *n* = 10 mice per group. Statistical analyses for subparts (B–D), (F), (H–J), and (L) were performed using one‐way ANOVA followed by Tukey’s post hoc test for multiple comparisons among treatment groups.  ^∗^ *p* < 0.05,  ^∗∗^ *p* < 0.01,  ^∗∗∗^ *p* < 0.001,  ^∗∗∗∗^ *p* < 0.0001; ns, not significant.

In the MagIC‐TI model, BL signals remained localized to the injected femur (*n* = 10 mice/group) (Figure 5A). Quantitative ROI analysis (Figure 5B), the percentage of GFP^+^ tumor cells in BM (Figure 5C, with the gating strategy shown in Figure S7), WT1 mRNA burden in BM (Figure 5D), and GFP expression in BM tissue sections (Figure 5E,F) all consistently demonstrated the therapeutic efficacy of HHT and DOX resistance. In contrast, within the IV model, BL signals were predominantly distributed in the lungs (unilaterally or bilaterally, as shown in Figure 5G). Although a decline in total tumor burden was observed in the HHT‐treated group by BLI, no statistically significant differences were found among the three treatment groups (*n* = 10 mice/group) (Figure 5H), and flow cytometry yielded similarly inconclusive results (Figure 5I, with gating strategy shown in Figure S8). WT1 expression in the lung was lower in the HHT group (*n* = 10 mice/group, *p*  < 0.05), but no significant intergroup differences were detected in the spleen or BM (*n* = 10/group, *p*  > 0.05) (Figure 5J). GFP expression in the BM tissue remained low across all IV groups (Figure 5K,L). Collectively, these findings demonstrate that the focal engraftment achieved in the MagIC‐TI model enables clear discrimination of drug sensitivity and resistance, whereas the disseminated, heterogeneous disease burden in the IV model obscures such assessments.

## 4. Discussion

The BM niche is a critical regulator of AML drug resistance and relapse, yet conventional preclinical models have failed to recapitulate this niche‐dependent pathophysiology with sufficient fidelity [17, 20–22]. The present study addresses this gap through the development of the MagIC‐TI model, which leverages magnetic targeting and precision intramedullary delivery to achieve rapid, localized engraftment of DOX‐resistant AML cells within the native BM microenvironment.

A principal advantage of the MagIC‐TI model is its ability to restrict leukemic engraftment primarily to the BM, thereby recapitulating the protective niche that drives MRD persistence and therapy resistance. The BM microenvironment is recognized to shield AML cells from chemotherapy and to contribute to relapse, yet conventional models fail to reproduce this niche‐dependent pathophysiology [7]. The fitness of an AML model has therefore been judged by its capacity to mirror the human pattern of BM‐predominant disease at early stages. In conventional IV xenograft models, leukemic cells disseminate early to extramedullary sites such as the lungs and spleen before establishing stable BM colonization, a phenomenon widely reported as a major limitation of IV‐based patient‐derived xenograft (PDX) approaches [8]. This stochastic, multiorgan distribution fails to reproduce the focal BM disease characteristic of human AML and has been shown to confound drug efficacy assessments. By contrast, the integrated bioluminescence, histopathological, flow cytometric, and molecular analyses collectively demonstrated that the MagIC‐TI model confines the tumor burden predominantly to the injected femur, whereas the IV model yields disseminated, multiorgan disease. This focal engraftment reduces tumor‐related complications under SPF conditions, prolongs survival, and widens the therapeutic window, thereby addressing the narrow observation window typical of disseminated models. Consistent with this, MagIC‐TI mice exhibited prolonged survival compared with the IV group, and when bioluminescence signal intensity exceeding 1 × 10^6^ photons/s was used as the treatment initiation threshold, the MagIC‐TI model permitted treatment as early as day 7 posttransplantation, whereas the IV model required a mean of 21 days to reach comparable tumor burden (Figure 3). This early, predictable engraftment also minimized interanimal variability and maximized the detectability of drug effects, enabling clearer discrimination between HHT sensitivity and DOX resistance, a distinction that was obscured by the heterogeneous and multifocal disease distribution in the IV model. Such heterogeneity has been highlighted as a critical source of interanimal variability and a major confounder in genetically engineered mouse models (GEMMs) and PDX‐based preclinical drug testing.

A prerequisite for the utility of any cell labeling strategy is that it does not fundamentally alter the biological properties of the target cells [23, 24]. In this regard, CD33 magnetic labeling had no significant impact on the immunophenotype, DOX resistance, morphology, proliferation, or apoptosis of HL60‐ADR‐GL cells. Flow cytometry confirmed stable and comparable surface expression of CD45 and CD33 in both MagRe and Non‐Mag cells (Figure 2A), and TEM (Figure 2B) revealed that the vast majority of MagRe cells retained intact ultrastructure, comparable to that of Non‐Mag cells. Consistently, CCK‐8 proliferation assays over 7 days (Figure 2C) and annexin V apoptosis monitoring over 28 days (Figure 2D) revealed no significant differences between the MagRe and Non‐Mag groups. Importantly, a control experiment without magnetic guidance further confirmed that CD33 magnetic labeling does not alter the intrinsic homing, engraftment, or systemic distribution of HL60‐ADR‐GL cells (Figures S9 and S10), ruling out labeling‐induced artifacts as a contributor to the improved engraftment efficiency observed in the MagIC‐TI system.

In the initial in vitro screening, targeted qPCR analysis of selected apoptosis‐related (BAX and CASP3), ferroptosis‐related (GPX4 and ACSL4), and homing‐related (CXCR4 and MAPK1) genes revealed no significant differences between MagRe and Non‐Mag cells after 48 h of culture in the absence of an external magnetic field (Figure 2E). These findings suggest that the MagIC‐TI strategy likely exerts its effects primarily through physical force rather than through molecular pathway alterations induced by magnetic labeling. However, this targeted panel represents only a preliminary screen, and a comprehensive, unbiased assessment of potential molecular alterations would require transcriptomic profiling at higher resolution, ideally by single‐cell RNA‐seq on donor cells retrieved directly from the BM of MagIC‐TI mice in future studies. Supporting the physical mechanism, in vitro migration assays demonstrated that MagRe cells, but not unlabeled cells, could undergo directional antigravity migration and Transwell migration under a magnetic field and assemble into multicellular clusters around the magnetic source (Figure 2F,G). These magnetic field‐driven behaviors are likely to facilitate the initial retention and colonization of magnetically labeled cells at the intramedullary injection site, contributing to the superior engraftment efficiency in vivo. Whether this physical confinement generates distinct mechanotransduction signals or niche interactions in more immunologically complete models warrants further exploration.

The MagIC‐TI model conferred a prolonged survival compared with the IV model, with the first death occurring on day 54 in the MagIC‐TI group versus day 46 in the IV group and with a markedly shorter engraftment time (Figure 3D). This combination of early, predictable engraftment and extended survival substantially widened the therapeutic window for drug efficacy evaluation. Throughout the 28‐day observation period, no significant differences in body weight were observed between the two groups, and hematological parameters, which included WBC, HGB, and PLT, did not differ significantly between the MagIC‐TI and IV groups at any time point examined. The mild elevation in WBC counts observed in both groups from day 8 postimplantation may be attributable to disease‐associated inflammatory responses and early tumor cell expansion (Figure 3F). However, flow cytometry and WT1 qPCR of peripheral blood mononuclear cells failed to detect significant circulating tumor burden at this stage, likely reflecting the dilution effect of the peripheral blood compartment, which may mask the low‐level leukemic cell presence.

The differential organ distribution of the tumor burden between the two models was consistently revealed across multiple detection modalities. In the IV model, in vivo bioluminescence imaging detected only pulmonary signals (Figure 3A), whereas ex vivo bioluminescence improved sensitivity by revealing signals in both the lungs and spleen (Figure 4A), though signals in the liver and kidney remained undetectable. Confocal fluorescence microscopy of tissue sections further identified GFP‐positive infiltrates in the lungs, spleen, liver, and kidney (Figure 4C), findings that were corroborated by elevated WT1 mRNA levels in these organs (Figure 4F). This gradient of detection sensitivity from in vivo bioluminescence to ex vivo bioluminescence to confocal microscopy indicates that direct fluorescence imaging of GFP is a more sensitive modality than bioluminescence for detecting low‐level leukemic infiltration and highlights the value of a multimodal readout strategy for comprehensive tumor burden assessment. In the MagIC‐TI model, by contrast, all modalities consistently pointed to focal femoral disease, underscoring the minimal systemic dissemination at this early time point.

The ability to visualize engrafted cells within the hard bone tissue represents an additional technical strength of this platform. By employing the SSD system, robust GFP fluorescence was preserved in fixed, decalcified BM sections from MagIC‐TI mice (Figure 4C), enabling direct confocal imaging of donor cells within the bone matrix. This integration of magnetic intramedullary delivery with SSD‐based fluorescence retention provides a powerful tool for investigating the spatial dynamics of drug action and resistance evolution within the BM niche.

The translational utility of the MagIC‐TI model for preclinical drug evaluation was demonstrated in the resistance study (Figure 5). With the same bioluminescence‐based treatment initiation criterion, testing could commence at day 7 in the MagIC‐TI model versus day 21 in the IV model. In the MagIC‐TI model, bioluminescence, flow cytometry, and WT1 qPCR in BM all consistently showed significant tumor burden reduction following HHT treatment and resistance to DOX. In the IV model, by contrast, only WT1 levels in the lung, but not in the spleen or BM, discriminated the HHT‐treated group from the controls. Bioluminescence and flow cytometry yielded inconclusive results across treatment groups. These findings demonstrate that focal BM engraftment simplifies and enhances the reliability of preclinical drug efficacy and resistance assessments.

Technologically, the MagIC‐TI model integrates three innovations CD33‐conjugated magnetic microbeads for cell targeting, a patented intramedullary injection system for precision delivery, and the SSD technique for preserving GFP fluorescence in BM tissues. The SSD system preserves GFP fluorescence for donor cell observation within the hard bone tissue, establishing a physiological context for studying minimal MRD persistence and niche‐dependent resistance mechanisms [25, 26]. This methodological advancement represents a significant step toward bridging the gap between in vitro drug screening and clinical outcomes.

Despite these advances, certain limitations of the current study should be acknowledged. First, the use of immunodeficient NSG mice precludes the investigation of immune‐leukemia interactions, which are increasingly recognized as critical to disease progression and therapy response [27–29]. Future studies employing humanized mouse models would help address this gap. Second, this study was conducted exclusively in male mice. Consequently, sex‐dependent effects on leukemia engraftment, progression, or drug response were not evaluated [18, 19]. Future studies should include both sexes to evaluate potential sex differences in niche‐mediated resistance. Third, while the magnetic labeling and targeting strategy proved effective for HL60 cells, its generalizability to primary AML samples or other leukemia subtypes requires further validation [30, 31]. Fourth, the targeted qPCR analysis performed on cultured cells represents an initial in vitro screening. A comprehensive, unbiased assessment of potential molecular effects of magnetic labeling, especially those influenced by the BM microenvironment, would require single‐cell RNA‐seq on donor cells isolated directly from the BM of MagIC‐TI mice, which we plan to undertake in future studies. Finally, the long‐term stability of magnetic retention and potential off‐target effects under extended magnetic field exposure warrant additional investigation.

## 5. Conclusion

In summary, the MagIC‐TI model, a magnetically guided intramedullary xenograft platform, was developed and validated. This model achieves rapid, localized, and sustained engraftment of drug‐resistant AML cells within the BM niche. Compared with conventional IV models, this system provides superior engraftment efficiency, BM‐restricted disease with reduced systemic dissemination, prolonged survival with an extended therapeutic window, and markedly improved capacity to discriminate drug sensitivity from resistance. The platform integrates CD33 magnetic labeling, a patented precision intramedullary injection system, and a SSD technique that preserves GFP fluorescence for high‐resolution BM imaging, thereby enabling robust and reproducible preclinical evaluation of niche‐directed therapies. The current limitations include the use of immunodeficient mice, the exclusive inclusion of male animals, the unvalidated generalizability to primary AML samples, and the preliminary nature of molecular characterization. These limitations outline clear directions for future work. Addressing them through humanized mouse models and comprehensive single‐cell multiomics profiling will further enhance the translational value of the MagIC‐TI system for elucidating mechanisms of niche‐mediated drug resistance and accelerating the development of novel therapeutic strategies.

**Nomenclature**
AML: Acute myeloid leukemia
BM: Bone marrow
MagIC‐TI: Magnetically targeted intramedullary
IV: Intravenous
HL60‐ADR‐GL: Doxorubicin‐resistant HL60 cells expressing eGFP and luciferase
MagRes‐HL60‐GL: Magnetized HL60‐ADR‐GL cells
DOX: Doxorubicin
HHT: Homoharringtonine
LSCs: leukemia stem cells
MDR: Multidrug resistance
MRD: Minimal residual disease
BLI: Bioluminescence imaging
TEM: Transmission electron microscopy
FACS: Fluorescence‐activated cell sorting
WT1: Wilms’ tumor 1
eGFP: Enhanced green fluorescent protein
NSG: Nonobese diabetic (NOD) Cg‐Prkdc^scid^ IL2rg^tm1Wjl^/SzJ
H&E: Hematoxylin and eosin staining
W&G: Wright–Giemsa staining
RT‐qPCR: Real‐time quantitative polymerase chain reaction
SSD: Semisolid decalcification system.

## Author Contributions

Qiusui Mai, Taosong Liu, Luxia Tang, Xiaojun Xu, and Qianli Jiang participated in the study design, analysis of data, and writing of the manuscript. Taosong Liu, Luxia Tang, Lihan Dai, Siyi Li, Jingyi Guo, Wen Xia, Lingxi Chen, Jiabao Wu, Jiawei Rong, Weishen Zhang, and Jiaxing Zhang performed the laboratory examination. Taosong Liu, Luxia Tang, and Qianli Jiang provided key materials. Qiusui Mai, Taosong Liu, Luxia Tang, Xiaojun Xu, and Qianli Jiang revised the manuscript.

## Funding

This work was supported by the grants from the Guangdong Basic and Applied Basic Research Foundation (Grants 2023A1515110236, 2018A030313647, and 2016A030313585), Research Start‐up Fund of Post‐doctoral of SAHSYSU (Grant ZSQYRSFPD0077) and the Innovation Program for Undergraduates of Southern Medical University (Grants 202412121329, 202412121343, and 202612121029).

## Disclosure

All authors read and approved the final version of manuscript.

## Ethics Statement

All animal studies were approved by the Experimental Animal Center of Southern Medical University (Ethical Approval Number IACUC‐LAC‐20241010‐007).

## Consent

Participants gave their permission for their names to be published.

## Conflicts of Interest

The authors declare no conflicts of interest.

## Supporting Information

Additional supporting information can be found online in the Supporting Information section.

## Supporting information


**Supporting Information** Supporting Information accompany this manuscript and provide additional methodological details and supporting data. Methods include the lentiviral transduction of HL60‐ADR cells, drug sensitivity assay and resistance fold calculation, H&E staining, W&G staining, and the in vivo homing dynamics of magnetically labeled cells without magnetic guidance. Results present validation data for lentiviral transduction efficiency (Figure S1), doxorubicin resistance of HL60‐ADR‐GL cells (Figure S2), phenotypic stability of magnetized cells (Figure S3), apoptosis analysis (Figure S4), histopathological and cytological evidence of leukemic infiltration patterns (Figure S5), gating strategies for flow cytometry in various experimental groups (Figures S6–S8), and the evaluation of magnetic labeling effects on in vivo homing and systemic distribution (Figures S9 and S10). Table S1 lists the primer sequences used for qPCR analysis. All figures and tables are referenced in the main text.

## Data Availability

Data in this research are available from the main text, and additional requests can be made to the corresponding authors.
