# Supplementary material for: A Novel Magnetically Targeted Intramedullary (MagIC‐TI) Xenograft Model for Precise Leukemia Modeling and Drug Resistance Evaluation in the Bone Marrow Niche
Source: J Immunol Res. 2026 Jun 28;2026:3236026. doi: 10.1155/jimr/3236026 (PMC13310384; doi:10.1155/jimr/3236026)
Supplement: Supplementary file 1 — Supporting Information Supporting Information accompany this manuscript and provide additional methodological details and supporting data. Methods include the lentiviral transduction of HL60‐ADR cells, drug sensitivity assay and resistance fold calculation, H&E staining, W&G staining, and the in vivo homing dynamics of magnetically labeled cells without magnetic guidance. Results present validation data for lentiviral transduction efficiency (Figure S1), doxorubicin resistance of HL60‐ADR‐GL cells (Figure S2), phenotypic stability of magnetized cells (Figure S3), apoptosis analysis (Figure S4), histopathological and cytological evidence of leukemic infiltration patterns (Figure S5), gating strategies for flow cytometry in various experimental groups (Figures S6–S8), and the evaluation of magnetic labeling effects on in vivo homing and systemic distribution (Figures S9 and S10). Table S1 lists the primer sequences used for qPCR analysis. All figures and tables are referenced in the main text. [file JIMR-2026-3236026-s001.docx]

# Supplementary Materials

# Supplementary Methods

### Lentiviral Transduction of HL60‑ADR Cells

The lentiviral vector co-expressing enhanced green fluorescent protein (eGFP) and firefly luciferase (plenti-eGFP-Luc) was constructed and stored in the laboratory of the Department of Hematology, Nanfang Hospital, Southern Medical University. HL60 cells were seeded at 1 × 10⁶ cells per well in 6-well plates and cultured overnight. The following day, the medium was replaced with fresh RPMI-1640 containing 8 μg/mL polybrene (Sigma-Aldrich, St. Louis, MO, USA). Lentiviral particles were produced by co-transfecting HEK293T cells with the plenti-eGFP-Luc vector and packaging plasmids (psPAX2 and pMD2.G) using Lipofectamine 3000 (Invitrogen, Carlsbad, CA, USA). Viral supernatant was harvested 48 h post‑transfection, filtered through a 0.45 μm filter, and added to HL60-ADR cells at a multiplicity of infection (MOI) of 10. After 24 h, the virus-containing medium was replaced with complete RPMI‑1640. Cells were cultured for an additional 72 h to allow transgene expression. To obtain a highly pure population of transduced cells, GFP‑positive cells were enriched by fluorescence‑activated cell sorting (FACS, BD FACSAria™ III, BD Biosciences). The sorted cells, designated HL60‑ADR‑GL, were expanded in complete medium. All transduction experiments were performed in triplicate.

### Drug Sensitivity Assay and Resistance Fold Calculation

HL60 and HL60‑ADR-GL cells in logarithmic growth phase were harvested and adjusted to a density of 1×10⁵ cells/mL. One hundred microliters of cell suspension were seeded per well into 96‑well plates and allowed to adhere for 2 h at 37 °C. HL60 cells were treated with doxorubicin (Sigma‑Aldrich, St. Louis, MO, USA) at concentrations of 0.01, 0.03, 0.10, 0.30 and 1.00 μg/mL, while HL60‑ADR‑GL cells were treated with 1.0, 3.0, 10.0, 30.0 and 100.0 μg/mL doxorubicin. Each concentration was tested in triplicate. Blank control wells (medium only) and negative control wells (cells without drug) were included. The final volume per well was adjusted to 200 μL. After 20 h of incubation, 20 μL of MTT solution (5 mg/mL, Sigma‑Aldrich) was added to each well, and the plates were incubated for an additional 4 h (total drug exposure time 24 h). The plates were then centrifuged at 1000 rpm for 5 min, the supernatant was carefully discarded, and 170 μL of dimethyl sulfoxide (DMSO) was added to each well. The plates were shaken gently to dissolve the formazan crystals. Absorbance was measured at 570 nm using a microplate reader (Bio‑Rad, Hercules, CA, USA). The cell proliferation inhibition rate was calculated as:

$$\text{Inhibition rate(\%)=}\left[ \text{1-}\frac{\text{OD}_{\text{treated}}\text{-}\text{OD}_{\text{blank}}}{\text{OD}_{\text{control}}\text{-}\text{OD}_{\text{blank}}} \right]\text{×100\%}$$

Dose‑response curves were generated by plotting the inhibition rate against the logarithm of doxorubicin concentration. The half‑maximal inhibitory concentration (IC₅₀) was calculated for each cell line using nonlinear regression (log[inhibitor] *vs*. normalized response-variable slope) in GraphPad Prism 9.0 (GraphPad Software, San Diego, CA, USA). The resistance fold was defined as the ratio of the IC₅₀ of HL60‑ADR‑GL cells to that of HL60 cells.

### Hematoxylin and eosin (H&E) staining and Wright-Giemsa (W&G) staining of Tissue Sections and Bone Marrow Smears

On day 21 post-injection, mice from the three groups (n=10 mice/group) were sacrificed by cervical dislocation, and lung, spleen, liver, kidney, and bone marrow were collected. The excised tissues were fixed in 4% paraformaldehyde, embedded in paraffin, and cut into 2-μm sections for H&E staining. For bone marrow, the injected femur was divided into two parts; bone marrow from one part was aspirated using a bone marrow aspiration needle to prepare smears, which were subsequently stained W&G stain. All stained sections and smears were examined under a light microscope (Olympus BX53; Olympus, Tokyo, Japan).

### In Vivo Homing Dynamics of MagRe and Non‑Mag Cells Without Magnetic Guidance

To determine whether CD33 magnetic labeling alters the bone marrow homing and retention capacity of HL60 cells, magnetically labeled MagRes‑HL60‑GL cells (MagRe group) or unlabeled HL60‑ADR‑GL cells (Non‑Mag group) were injected intramedullarily into the right femur of NSG mice (5×10⁶ cells/30 μL, n=10 mice per group) without application of an external magnetic field. The cell preparation and intramedullary injection procedure (patented micro‑injection needle, Patent No. CN201620090904.2) were identical to those described in the main Methods. Bioluminescence imaging (BLI) was performed on days 0, 7, 14, 21, and 28 post‑injection using an IVIS system (exposure time 60 s) following intraperitoneal injection of D‑luciferin (100 μL, 10 mg/mL).

# Supplementary Results

### Lentiviral Transduction of HL60‑ADR Cells with eGFP and Luciferase

Flow cytometric analysis performed 72 hours post-transduction revealed that 90.2%±2.1% (mean±SD, n=5 replicates) of the cells expressed eGFP (Fig. S1 A, B). Following FACS enrichment, the purity of GFP⁺ cells exceeded 95% (Fig. S1 C, D). The transduced and sorted cells were designated HL60-ADR-GL.

### Doxorubicin resistance of HL60‑ADR‑GL cells

The MTT assay was used to compare the doxorubicin sensitivity between HL60 and HL60‑ADR‑GL cells. As shown in Fig. S2, doxorubicin inhibited the proliferation of both cell lines in a concentration‑dependent manner. The IC₅₀ values derived from the dose‑response curves were 0.091 ± 0.009 μg/mL for HL60 cells and 8.07±0.88 μg/mL for HL60‑ADR‑GL cells (mean±SD, n= 5 replicates). The calculated resistance fold was 88.7, indicating that HL60‑ADR‑GL cells are highly resistant to doxorubicin.

### H&E and W&G staining reveal distinct patterns of leukemic infiltration

In the MagIC-TI group, histopathological examination revealed no or only minimal suspicious extramedullary leukemic infiltration in the examined organs (Fig. S5A–D), suggesting that the disease was largely confined to the bone marrow during the early phase of progression. W&G staining of bone marrow smears provided further cytological evidence: smears from the MagIC-TI group were hypercellular and dominated by a homogeneous population of cells with blast morphology, suspicious for leukemic blasts (Fig. S5E). In contrast, bone marrow smears from the IV group at the same time point retained a relatively normal morphological appearance (Fig. S5J), consistent with primary involvement of peripheral organs rather than the bone marrow, as evidenced by H&E staining that demonstrated areas suspicious for extensive leukemic infiltration in the lung (Fig. S5F), spleen (Fig. S5G), and liver (Fig. S5H), whereas suspicious infiltration in the kidney was not prominent (Fig. S5I).

### Magnetic Labeling Does Not Affect In Vivo Homing or Systemic Distribution After Intramedullary Injection Without Magnetic Guidance

Immediately after intramedullary injection, bioluminescence imaging (BLI) revealed tumor signals restricted to the injected right femur in both the MagRe and Non‑Mag groups (n=10 mice per group) (Fig. S9A). On days 7 and 14 post‑injection, no BLI signals were detected, indicating that the transplanted cells did not proliferate during this period. Tumor signals reappeared on day 21, initially in the lungs; by day 28, scattered signals were observed in both the injected femur and the lungs. No significant differences in BLI signal intensity or distribution were observed between the MagRe and Non‑Mag groups(*P*>0.05, n=10 mice/group) (Fig. S9B). Flow cytometric analysis on day 21 showed an increased proportion of GFP⁺ cells in the bone marrow of both the MagRe and Non‑Mag groups compared with the negative control group (MagRe vs. CTRL: *P*<0.01; Non-Mag *vs*. CTRL: *P*<0.05, n=10 mice per group) (Fig. S9C, with gating Strategy showed in Fig. S10), although the positive population was not distinctly separated; no significant difference was detected between the two groups (*P*>0.05, n=10 mice/group) (Fig. S9C). In peripheral blood, no increase in GFP⁺ cells were detected in either group. RT‑qPCR analysis on day 21 revealed detectable WT1 mRNA expression in the lung, spleen, bone marrow, and peripheral blood. WT1 levels in both the MagRe and Non‑Mag groups were elevated relative to the control group (MagRe or Non-Mag vs. CTRL: *P*<0.0001, n=10 mice /group, Fig. S10D), but no significant difference was observed between the two groups (*P*>0.05, n=10 mice/group) (Fig. S9D). Together, these findings indicate that magnetic labeling of HL60‑ADR‑GL cells does not significantly alter their in vivo homing, engraftment, or systemic distribution following intramedullary injection.

# Supplementary Tables.

### Supplementary Table S1. Primer sequences for qPCR analysis.

| **Gene** | **Forward Primer (5‘→3’)** | **Reverse Primer (5’→3‘)** | **Pathway** |
| --- | --- | --- | --- |
| BAX | CTCAAGGCCCTGTGCACTAAA | CCCGGAGGAAGTCCAGTGT | Apoptosis (pro‑apoptotic) |
| CASP3 | TGGAATGTCATCTCGCTCTGGT | CTGAAAGGGACTGGATGAACC | Apoptosis (effector caspase) |
| GPX4 | GAGGCAAGACCGAAGTAAACTAC | CCGAACTGGTTACACGGGAA | Ferroptosis (suppressor) |
| ACSL4 | ATATTCGTCACCACTCACA | CTTCACGTACTCCACATCCCC | Ferroptosis (promoter) |
| CXCR4 | GAGGGGATCAGTATATACACTTCAG | CGATGAGGACATGGACAAGG | Homing receptor |
| MAPK1 | TACAAGATCTGTATTCCTGGG | AGCATCTGGGGAAGGAAATC | MAPK signaling |
| β‑actin | CACGAAACTACCTTCAACTCCATC | AGCACTGTGTTGGCGTACAG | Internal control |

# Supplementary Figures.


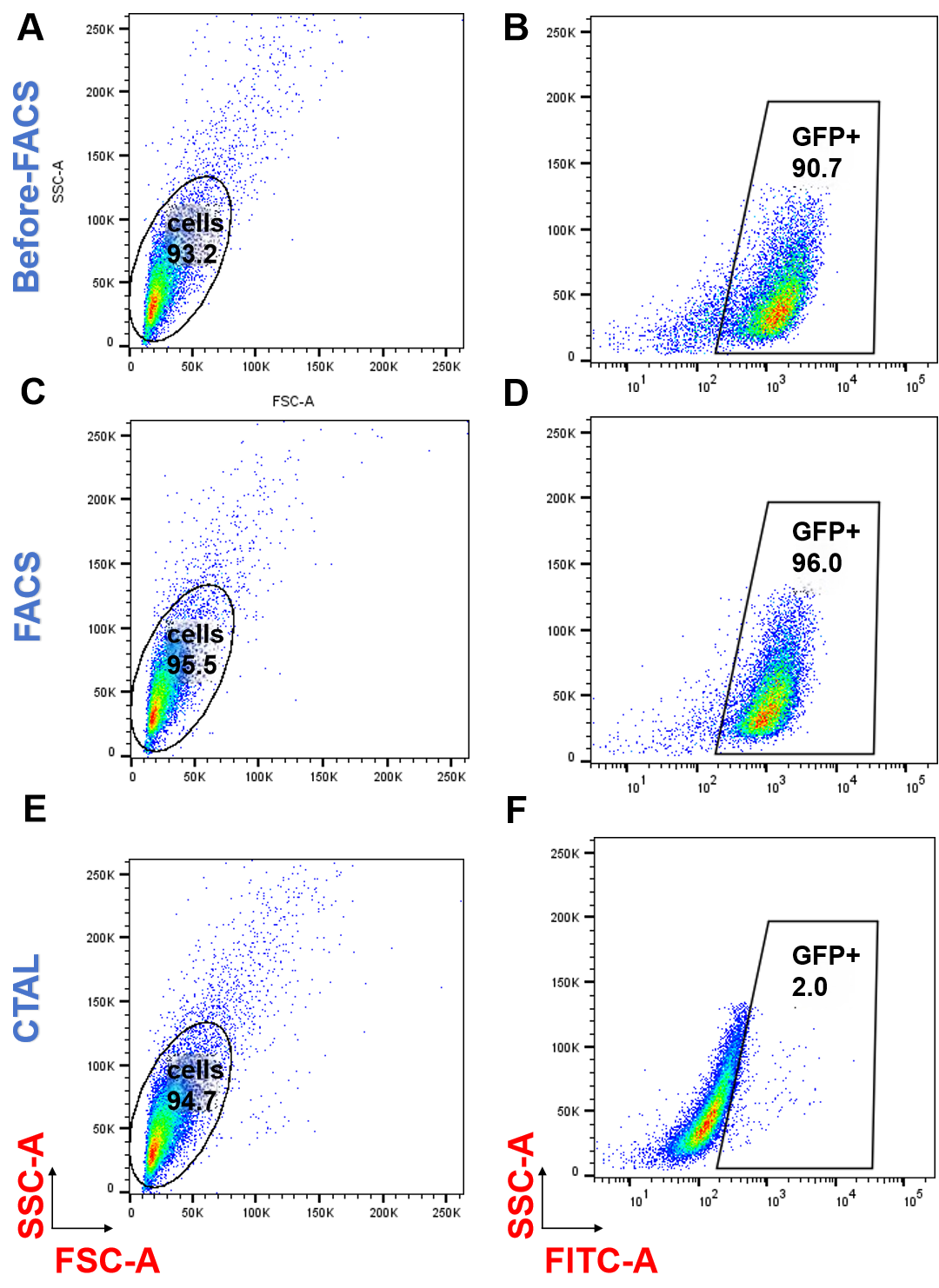


### Fig. S1. Gating strategy for the analysis of GFP expression following lentiviral transduction and FACS enrichment

(A, B) Flow cytometric analysis of HL60-ADR cells at 72 h post‑transduction with plenti‑eGFP‑Luc lentivirus. (C, D) Analysis of the same cell population after FACS enrichment of GFP^+^ cells. (E, F) Untransduced HL60-ADR cells serving as the negative control for defining the GFP^+^ gate. (A, C, E) The cell population of interest was first identified and gated based on forward scatter (FSC) and side scatter (SSC) parameters to exclude debris and non-viable cells. (B, D, F) GFP fluorescence intensity was subsequently assessed in the gated viable singlet population. The GFP-positive gate was set using untransduced HL60-ADR cells shown in (E) and (F). The mean transduction efficiency before sorting was 90.2±2.1％（n = 5 replicates), and the purity of GFP⁺ cells after sorting was 96.28±3.24％（n = 5 replicates). Data information: Representative plots from one of five replicates are shown. Quantitative values are presented as mean±SD.

### Fig. S2. Doxorubicin sensitivity of HL60 and HL60-ADR-GL cells.

Dose-response curves of parental HL60 cells and doxorubicin-resistant HL60-ADR-GL cells treated with increasing concentrations of doxorubicin for 24 h. Cell proliferation inhibition rate was determined by MTT assay. Data points represent the mean proliferation inhibition rate (%) ± SD from five replicates. Half-maximal inhibitory concentration (IC_50_) values were calculated by nonlinear regression (log[inhibitor]*vs*. normalized response-variable slope) in GraphPad Prism 9.0, yielding 0.091 ± 0.009 μg/mL for HL60 cells and 8.07 ± 0.88 μg/mL for HL60-ADR-GL cells. The resistance fold, defined as the ratio of the two IC_50_ values, was 88.7, confirming high-level doxorubicin resistance. Data information: All quantitative data are presented as mean±SD with n = 5 replicates. IC_50_ values are reported as mean±SD.


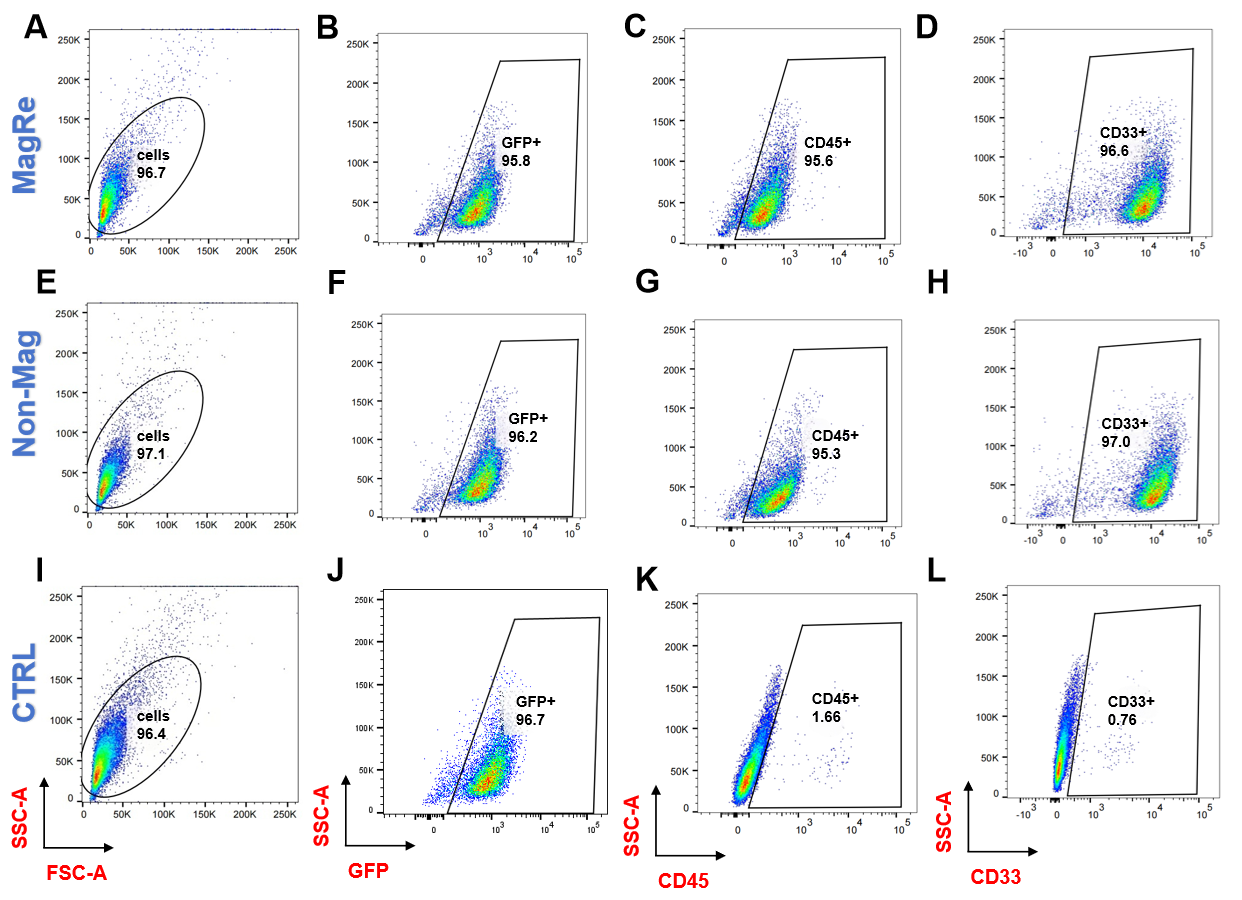


### Fig. S3. Gating strategy for the analysis of phenotypic stability of magnetized HL60-ADR-GL cells.

(A-D) Magnetically labeled MagRe cells. (E-H) Unlabeled Non-Mag cells. (I-L) Unstained negative control cells (no CD45-APC or CD33-PerCP-Cy5.5). (A, E, I) The cell population of interest was first identified and gated based on forward scatter (FSC) and side scatter (SSC) parameters to exclude debris and non-viable cells. (B, F, J) GFP fluorescence was assessed in the gated viable population to identify successfully transduced GFP^+^ cells. (C, G, K) CD45-APC expression was analyzed within the GFP^+^ gate. (D, H, L) CD33-PerCP-Cy5.5 expression was analyzed within the GFP gate. The positive gates for CD45 and CD33 were set using the unstained negative control cells shown in (K) and (L). The percentages of CD45^+^ and CD33^+^ cells within the GFP^+^ population are indicated in the respective plots. Both MagRe and Non-Mag cells maintained high and stable surface expression of CD45 and CD33, confirming that the magnetic labeling process did not alter the immunophenotype of HL60-ADR-GL cells. Data information: Representative flow cytometry plots from one of five replicates are shown. Quantitative values of CD45 and CD33^+^ percentages are presented as mean±SD (n=5 replicates) in the main text.


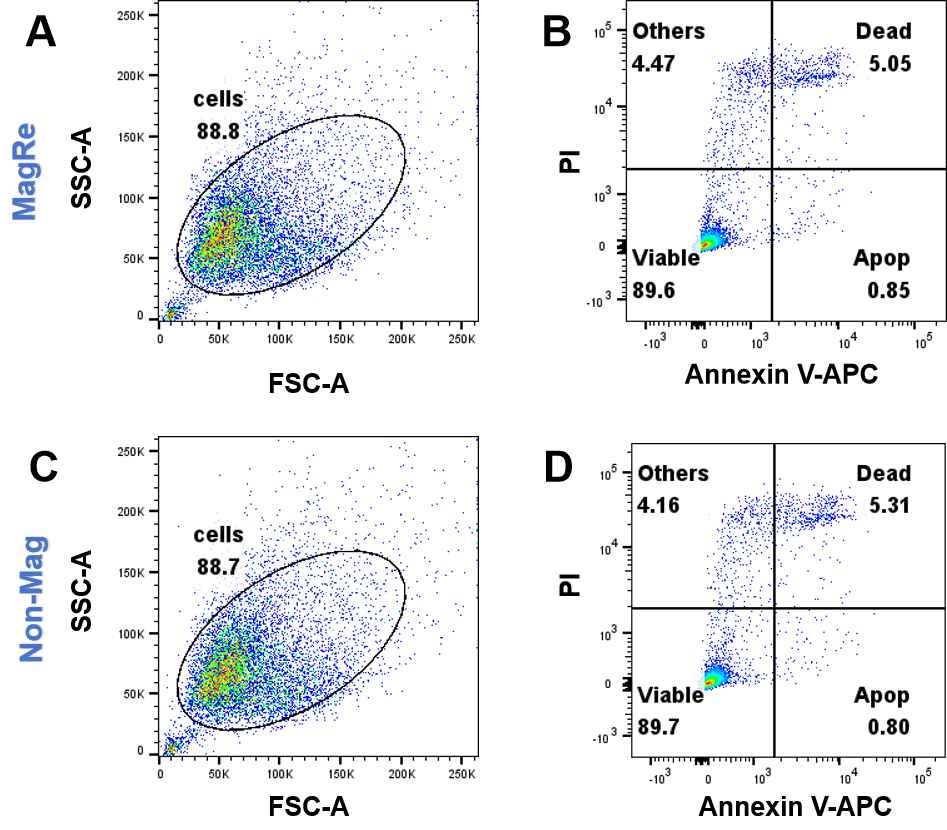


### Fig. S4. Gating strategy for the analysis of cell apoptosis by Annexin V-APC/PI staining.

(A, B) Magnetically labeled MagRe cells. (C, D) Unlabeled Non-Mag cells, serving as the untreated negative control for setting quadrant gates. (A, C) The cell population was first gated based on FSC and SSC parameters to exclude debris. (B, D) Apoptosis was assessed in the gated population using Annexin V-APC and PI staining. Quadrant gates distinguish viable (Annexin V⁻/PI⁻), early apoptotic (Annexin V⁺/PI⁻), and late apoptotic/necrotic (Annexin V⁺/PI⁺) cells. The percentage of apoptotic cells remained low and comparable between groups throughout the culture period, indicating that magnetic labeling did not induce apoptosis. Data information: Quantitative values of Annexin V⁺ cells are presented as mean±SD in the main text (n=5 replicates).


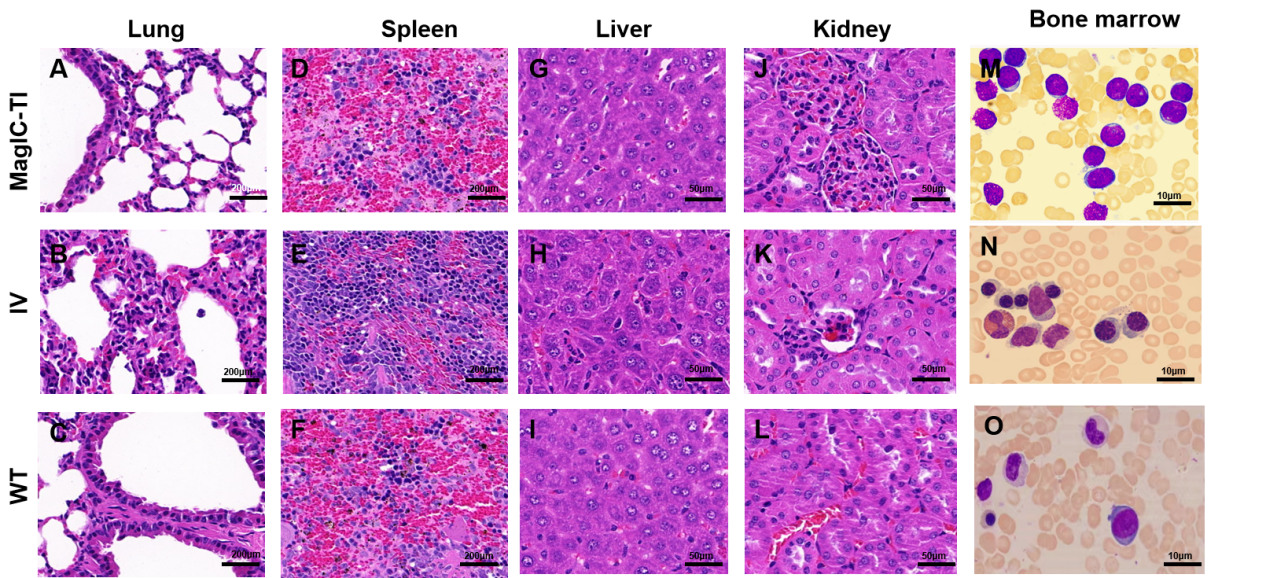


### Fig. S5. H&E staining of multiple organs and Wright-Giemsa staining of bone marrow smears in MagIC-TI, IV, and WT groups at day 21 post-transplantation.

(A-D) H&E staining of lung (A), spleen (B), liver (C), and kidney (D) from the MagIC-TI group. (E) Wright-Giemsa staining of bone marrow smear from the MagIC-TI group. (F–I) H&E staining of lung (F), spleen (G), liver (H), and kidney (I) from the IV group. (J) Wright-Giemsa staining of bone marrow smear from the IV group. In the MagIC-TI group, H&E staining revealed no or only minimal suspicious extramedullary leukemic infiltration in the examined organs (A–D), while Wright-Giemsa staining showed hypercellular bone marrow dominated by a homogeneous population of cells with blast morphology, suspicious for leukemic blasts (E). In contrast, the IV group exhibited areas suspicious for extensive leukemic infiltration in the lung (F), spleen (G), and liver (H), whereas suspicious infiltration in the kidney was not prominent (I). Bone marrow smears from the IV group retained a relatively normal morphological appearance (J), consistent with primary involvement of peripheral organs rather than the bone marrow. These distinct patterns confirm that the MagIC-TI model achieves localized bone marrow engraftment, whereas the IV model results in disseminated extramedullary disease. n=10 mice per group.


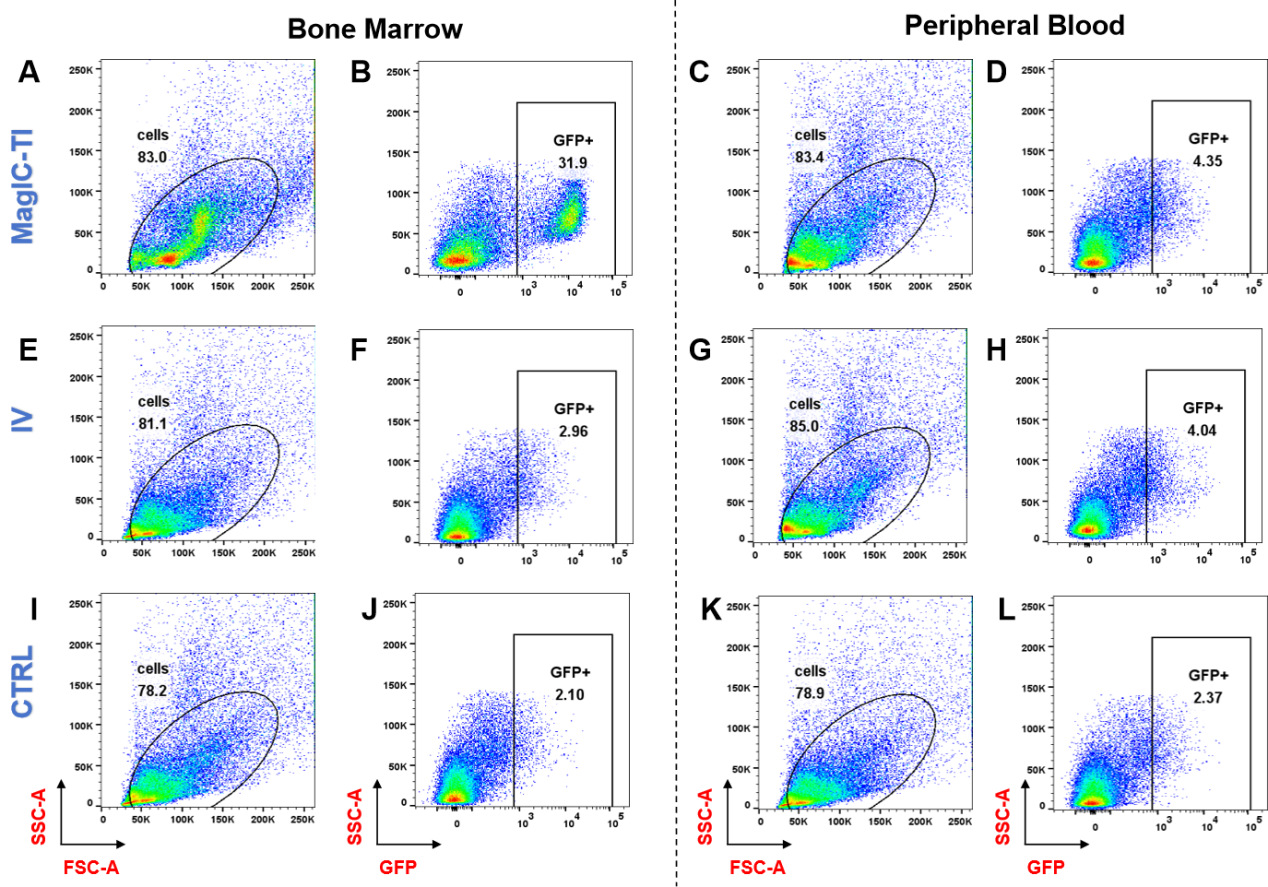


### Fig. S6. Gating strategy for flow cytometric analysis of GFP^+^ tumor cells in the injected femoral bone marrow and peripheral blood between MagIC-TI and IV.

(A-D) MagIC-TI group. (E-H) IV group. (I-L) WT group. (A, B) MagIC-TI femoral bone marrow. (C, D) MagIC-TI peripheral blood. (E, F) IV femoral bone marrow. (G, H) IV peripheral blood. (I, J) WT femoral bone marrow. (K, L) WT peripheral blood. (A, E, I, C, G, K) The cell population was first gated based on FSC and SSC parameters to exclude debris. (B, F, J, D, H, L) GFP expression was assessed in the gated population to identify tumor cells. A distinct GFP^+^ population was clearly detected in the femoral bone marrow of the MagIC-TI group, confirming successful local engraftment. Data information: Quantitative values of GFP^+^ cell percentages are presented as mean±SD in the main text (n=10 mice per group).


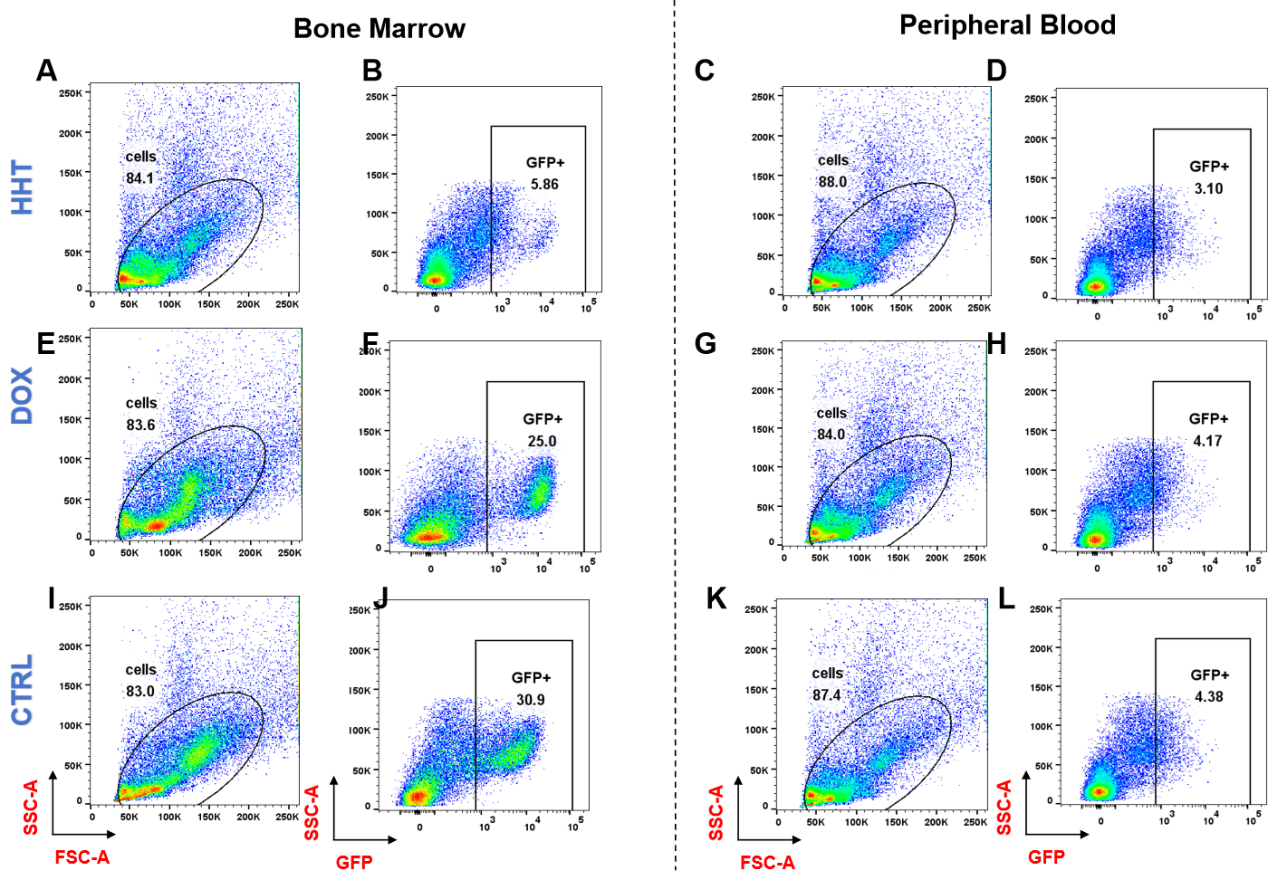


### Fig. S7. Gating strategy for flow cytometric analysis of GFP⁺ tumor cells in the injected femoral bone marrow and peripheral blood in the drug resistance evaluation assay using the MagIC-TI model.

(A-D) HHT group. (E-H) DOX group. (I-L) Ctrl group. (A, B) HHT femoral bone marrow. (C, D) HHT peripheral blood. (E, F) DOX femoral bone marrow. (G, H) DOX peripheral blood. (I, J) Ctrl femoral bone marrow. (K, L) Ctrl peripheral blood. (A, E, I, C, G, K) The cell population was first gated based on FSC and SSC parameters to exclude debris. (B, F, J, D, H, L) GFP expression was assessed in the gated population to identify tumor cells. A marked reduction in the GFP^+^ population was observed in the femoral bone marrow of the HHT group, whereas the DOX group retained a distinct GFP^+^ population, consistent with doxorubicin resistance. Data information: Quantitative values of GFP⁺ cell percentages are presented as mean±SD in the main text (n=10 mice per group).


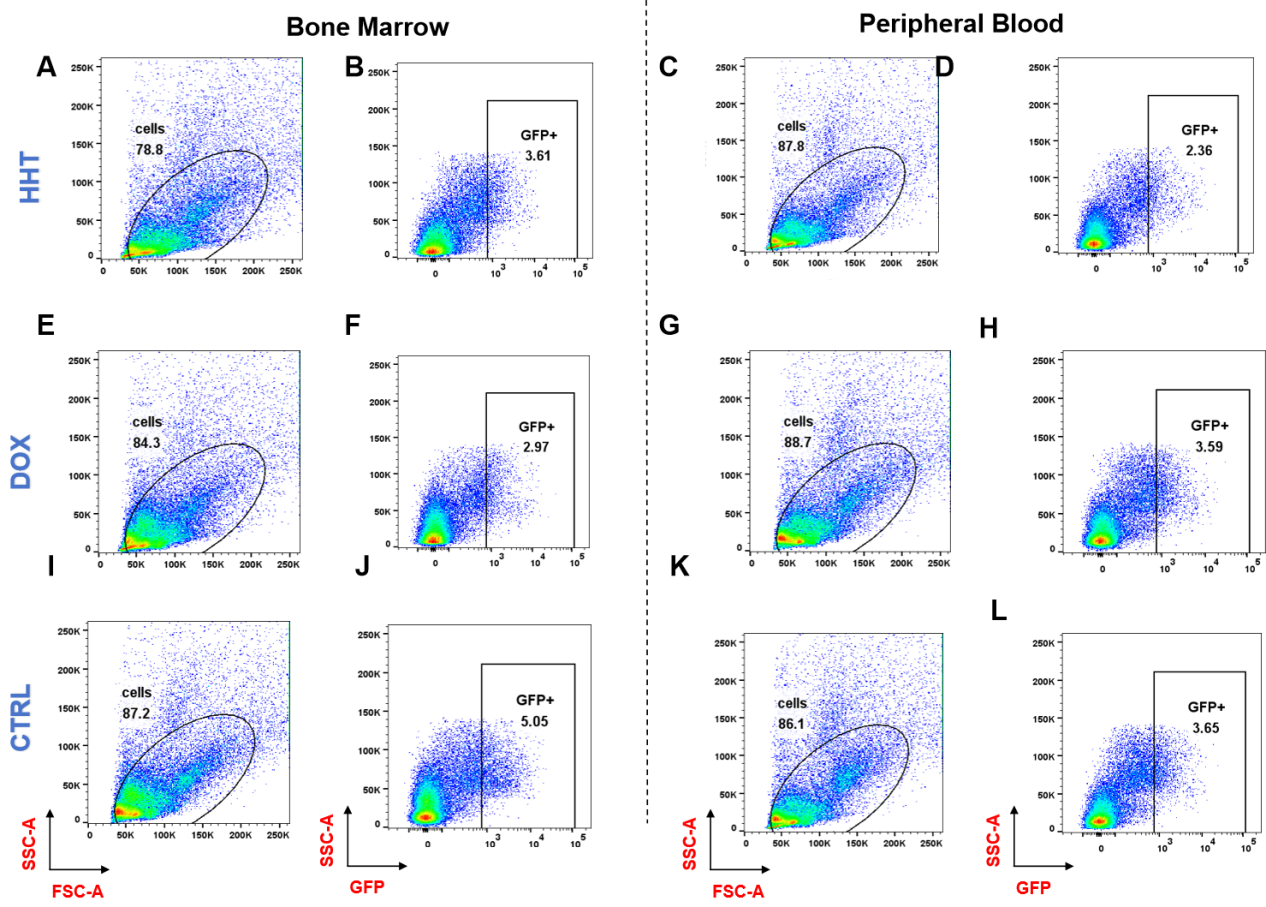


### Fig. S8. Gating strategy for flow cytometric analysis of GFP⁺ tumor cells in the injected femoral bone marrow and peripheral blood in the drug resistance evaluation assay using the IV model.

(A-D) HHT group. (E-H) DOX group. (I-L) Ctrl group. (A, B) HHT femoral bone marrow. (C, D) HHT peripheral blood. (E, F) DOX femoral bone marrow. (G, H) DOX peripheral blood. (I, J) Ctrl femoral bone marrow. (K, L) Ctrl peripheral blood. (A, E, I, C, G, K) The cell population was first gated based on FSC and SSC parameters to exclude debris. (B, F, J, D, H, L) GFP expression was assessed in the gated population to identify tumor cells. No significant differences in the GFP^+^ population were observed among the HHT, DOX, and Ctrl groups in the IV model. Data information: Quantitative values of GFP^+^ cell percentages are presented as mean ± SD in the main text (n = 10 mice per group).


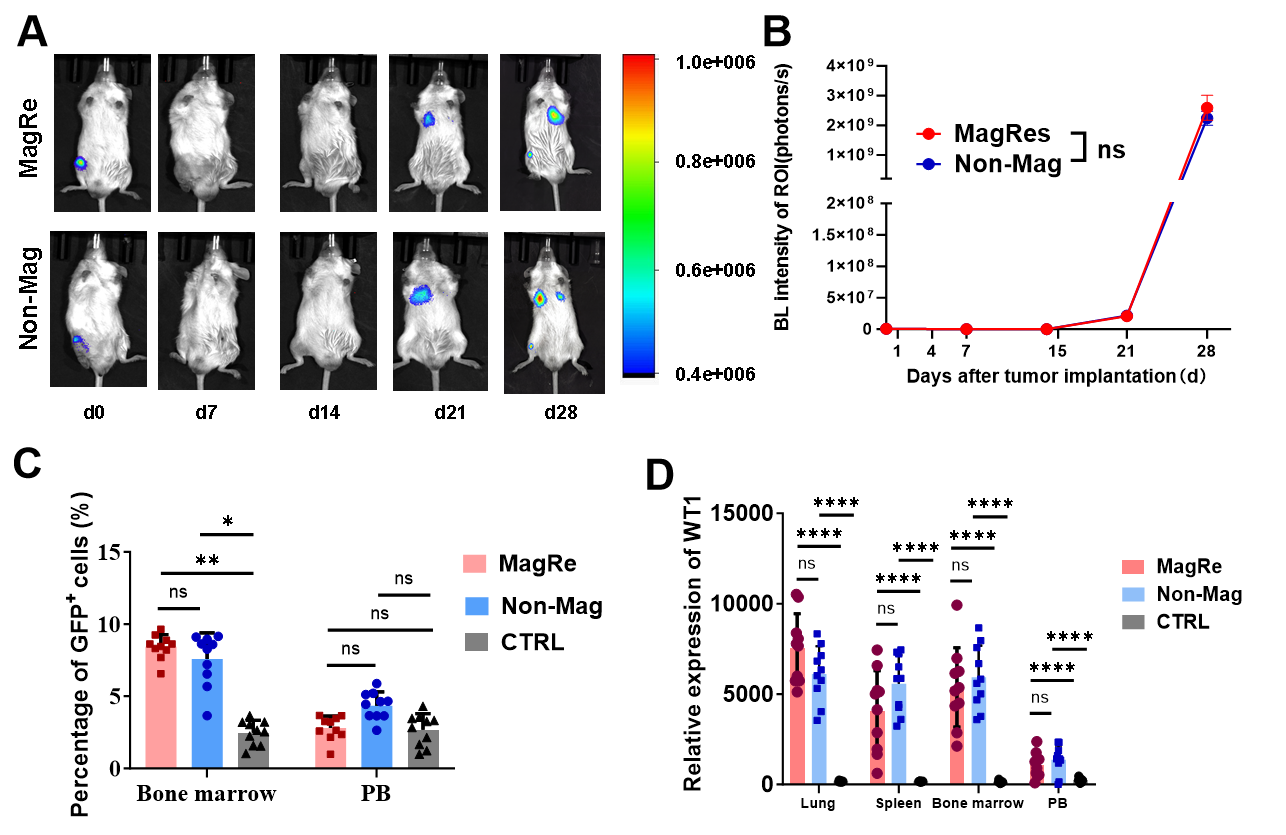


### Fig. S9. Magnetic labeling does not affect in vivo homing or systemic distribution after intramedullary injection without magnetic guidance.

(A) Representative bioluminescence images of MagRe and Non-Mag groups at days 0, 7, 14, 21, and 28 post-injection. (B) Quantification of BLI signal intensity in the region of interest over time. (C) Flow cytometric analysis of GFP⁺ cells in femoral bone marrow and peripheral blood on day 21 (gating strategy shown in Fig. S10). (D) WT1 mRNA expression in lung, spleen, bone marrow, and peripheral blood measured by RT-qPCR on day 21. BLI signals were initially restricted to the injected femur in both groups and reappeared on day 21, with no significant differences in signal intensity or distribution between groups (P > 0.05). An increased proportion of GFP⁺ cells was observed in the bone marrow of both MagRe and Non-Mag groups compared with the Ctrl group, but no significant difference was detected between the two groups. WT1 levels were elevated in both MagRe and Non-Mag groups relative to the Ctrl group, with no significant difference between the two groups. Together, these findings indicate that CD33 magnetic labeling does not alter the in vivo homing, engraftment, or systemic distribution of HL60-ADR-GL cells. Data are presented as mean±SD (n=10 mice/group). Statistical analysis: For (B), two-way repeated-measures ANOVA was used for between-group comparisons over time. For (C) and (D), one-way ANOVA followed by Tukey’s post hoc test was applied for comparisons among MagRe, Non-Mag, and Ctrl groups. *P* values are indicated in the main text.


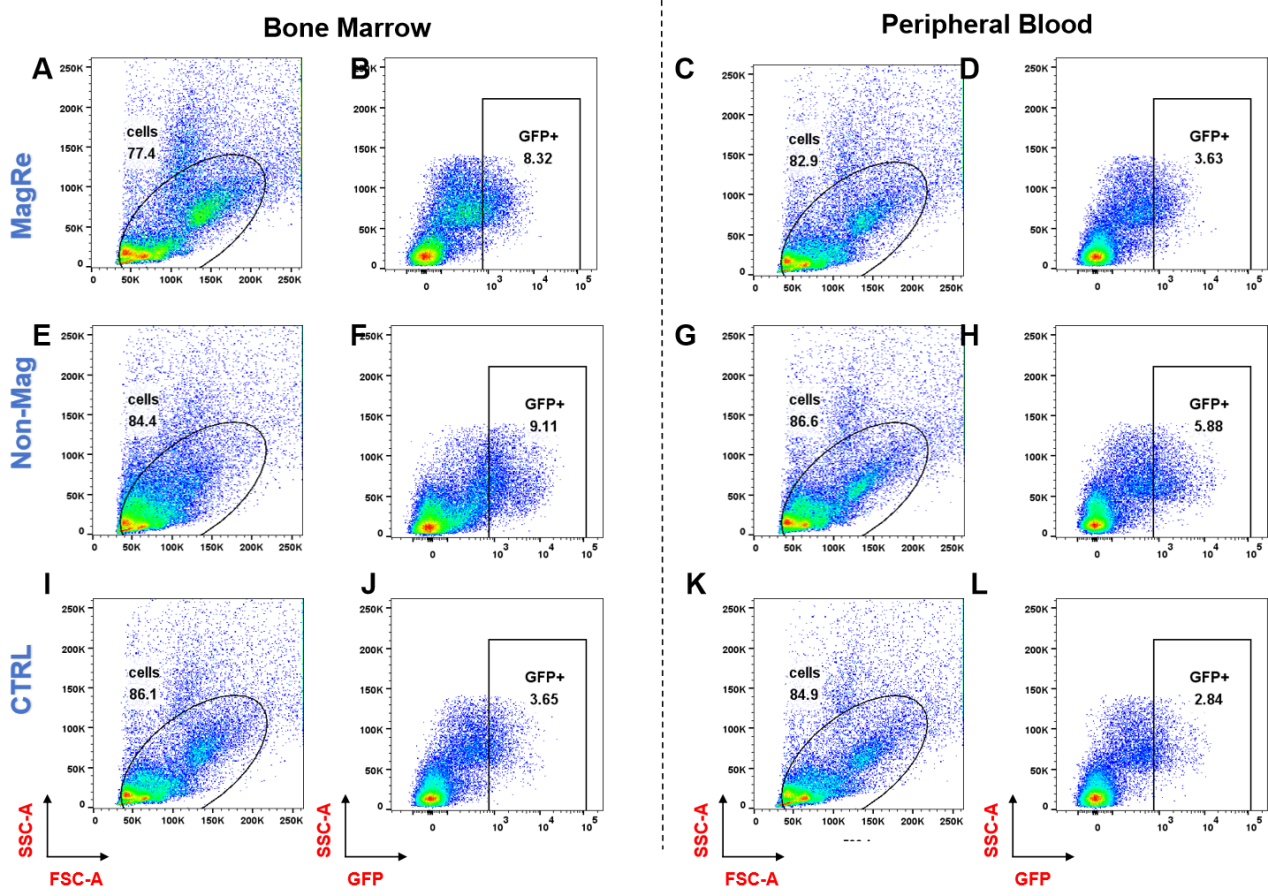


### Fig. S10. Gating strategy for flow cytometric analysis of GFP⁺ tumor cells in the injected femoral bone marrow and peripheral blood between MagRe and Non-Mag groups after intramedullary injection without magnetic guidance.

(A-D) MagRe group. (E–H) Non-Mag group. (I–L) Ctrl group. (A, B) MagRe femoral bone marrow. (C, D) MagRe peripheral blood. (E, F) Non-Mag femoral bone marrow. (G, H) Non-Mag peripheral blood. (I, J) Ctrl femoral bone marrow. (K, L) Ctrl peripheral blood. (A, E, I, C, G, K) The cell population was first gated based on FSC and SSC parameters to exclude debris. (B, F, J, D, H, L) GFP expression was assessed in the gated population to identify tumor cells. An increased proportion of GFP⁺ cells was observed in the bone marrow of both MagRe and Non-Mag groups compared with the Ctrl group, but no significant difference was detected between the two groups. These findings indicate that magnetic labeling does not alter the in vivo homing or engraftment capacity of HL60-ADR-GL cells. Quantitative values of GFP⁺ cell percentages are presented as mean±SD in the main text (n=10 mice/group).
